# Characterization of *Legionella pneumophila* Populations by Multilocus Variable Number of Tandem Repeats (MLVA) Genotyping from Drinking Water and Biofilm in Hospitals from Different Regions of the West Bank

**DOI:** 10.3390/pathogens9110862

**Published:** 2020-10-22

**Authors:** Ashraf R. Zayed, Marina Pecellin, Alaa Salah, Hanna Alalam, Suha Butmeh, Michael Steinert, Rene Lesnik, Ingrid Brettar, Manfred G. Höfle, Dina M. Bitar

**Affiliations:** 1 Department of Vaccinology and Applied Microbiology, Helmholtz Centre for Infection Research (HZI), Inhoffenstrasse 7, 38124 Braunschweig, Germany; ashraf_manfan@me.com (A.R.Z.); marinapecellin@gmail.com (M.P.); rene.lesnik@helmholtz-hzi.de (R.L.); ibrettar@web.de (I.B.); 2 Department of Microbiology and Immunology, Al-Quds University, Abu-Dies, East Jerusalem 19356, Palestine; alaa.hisham@uks.eu (A.S.); hanna.alalam@gu.se (H.A.); salbutmeh@staff.alquds.edu (S.B.); dbitar@staff.alquds.edu (D.M.B.); 3 Department of Life Sciences, Institute of Microbiology, Technical University of Braunschweig, Universitätsplatz 2, 38106 Braunschweig, Germany; m.steinert@tu-bs.de

**Keywords:** MLVA-genotypes, clonal complex, hospital water, West Bank, *Legionella pneumophila*

## Abstract

The West Bank can be considered a high-risk area for Legionnaires’ disease (LD) due to its hot climate, intermittent water supply and roof storage of drinking water. *Legionella*, mostly *L. pneumophila*, are responsible for LD, a severe, community-acquired and nosocomial pneumonia. To date, no extensive assessment of *Legionella* spp and *L. pneumophila* using cultivation in combination with molecular approaches in the West Bank has been published. Two years of environmental surveillance of *Legionella* in water and biofilms in the drinking water distribution systems (DWDS) of eight hospitals was carried out; 180 *L. pneumophila* strains were isolated, mostly from biofilms in DWDS. Most of the isolates were identified as serogroup (Sg) 1 (60%) and 6 (30%), while a minor fraction comprised Sg 8 and 10. Multilocus Variable number of tandem repeats Analysis using 13 loci (MLVA-8(12)) was applied as a high-resolution genotyping method and compared to the standard Sequence Based Typing (SBT). The isolates were genotyped in 27 MLVA-8(12) genotypes (Gt), comprising four MLVA clonal complexes (VACC 1; 2; 5; 11). The major fraction of isolates constituted Sequence Type (ST)1 and ST461. Most of the MLVA-genotypes were highly diverse and often unique. The MLVA-genotype composition showed substantial regional variability. In general, the applied MLVA-method made it possible to reproducibly genotype the isolates, and was consistent with SBT but showed a higher resolution. The advantage of the higher resolution was most evident for the subdivision of the large strain sets of ST1 and ST461; these STs were shown to be highly pneumonia-relevant in a former study. This shows that the resolution by MLVA is advantageous for back-tracking risk sites and for the avoidance of outbreaks of *L. pneumophila.* Overall, our results provide important insights into the detailed population structure of *L. pneumophila*, allowing for better risk assessment for DWDS.

## 1. Introduction

*Legionella* are opportunistic pathogens with a widespread distribution in freshwater environments. This bacterial genus is well known to cause legionellosis. The term “legionellosis” describes both Legionnaires’ disease (LD), a severe form of atypical pneumonia, and a nonpneumonic febrile illness called Pontiac Fever. Due to the low concentrations of nutrients in their aquatic habitats, *Legionella* have adapted to live in biofilms, where they can obtain amino acids and carbon sources that they need for survival, replication and protection from temperature changes and biocide treatment [1]. In biofilms, *Legionella* are part of complex microbial communities [2] where they are subjected to predation by protozoa [3]. The transmission of bacteria from the environment to humans occurs via inhalation or aspiration of *Legionella*-containing aerosols [4,5]. Among the more than 60 species of the genus *Legionella*, *L. pneumophila* is responsible for approximately 90% of all globally reported community- and hospital-acquired cases of legionellosis [6,7,8]. *L. pneumophila* has 15 serogroups (Sgs); Sg1 is the most common, causing LD. Sg6 comes second, and is also a causative agent of LD [9,10]. 

Many studies have demonstrated that the main sources for LD are the drinking water distribution systems (DWDS) in large buildings like hospitals and hotels [11,12,13]. The contamination of hospital water systems with *Legionella* is considered to pose a high risk for patients, especially for those with severe diseases. To this end, it is well known that LD is an important cause of hospital-acquired pneumonia [8]. The presence of *Legionella* in DWDS is a serious health risk to hospital staff and patients, but the magnitude of the problem is often unrecognized [8,14,15]. A high seroprevalence of *L. pneumophila* has been observed among health care workers [16]. The problem is compounded in the West Bank because awareness about the prevalence of *L. pneumophila* or LD is lacking, and few data are available for such arid regions. Furthermore, there are no specific guidelines for *L. pneumophila* surveillance or protection from exposure in hospitals or public buildings. 

Cultivation Dependent Analysis (CDA) on specific agar plates is the standard and recommended technique used for environmental surveillance of *L. pneumophila* [17]. One major obstacle in the isolation and quantification of *L. pneumophila* by culture is often its Viable But Non Culturable (VBNC) state, and overgrowth by competing bacteria [18,19,20]. On the other hand, Cultivation Independent Analyses (CIA) using PCR-based molecular approaches are rapid, sensitive and widely applied for the detection and identification of *L. pneumophila* [21]. Because of the widespread occurrence of *L. pneumophila* in large, man-made, freshwater systems, study of environmental isolates is needed for the implementation of prevention measures, and to identify possible sources of infection [22,23].

For the identification of possible sources of contamination/infection, high resolution genotyping of new isolates is needed to correlate environmental isolates with clinical isolates. This is currently done by two molecular approaches: Sequence Based Typing (SBT) and Multilocus Variable Number of Tandem Repeats (VNTR) Analysis (MLVA). SBT of *L. pneumophila* is done by sequencing a set of seven reference genes per isolate, providing a specific Sequence Type (ST) that can be matched with an International database [24]. MLVA has been widely used to identify different pathogens [25,26,27]. VNTRs consist of relatively short DNA fragments repeated in tandem that can vary in copy number among strains. For *L. pneumophila*, MLVA has exhibited an excellent ability to distinguish among strains if 8 to 12 different loci are used. It can be performed in a multiplexed manner, followed by capillary electrophoresis, enabling automated sample analysis and data acquisition. These advantages reduce typing time and costs. Several studies have used MLVA for the genotyping of *L. pneumophila* strains [28,29]. They showed the high correspondence between MLVA genotypes and STs with an important increase in resolution when applying MLVA, which is relevant for understanding clonal populations. Recent publications showed that the majority of clinically relevant strains were distributed into a limited number of Clonal Complexes (CCs) defined by MLVA, called VNTR analysis CC (VACC) and characterized by epidemic reference strains such as Paris (VACC1) and Philadelphia-1 (VACC2) [29]. Due to its advantages, MLVA could complement SBT for large sets of isolates and enable insights into the clonal structure of *L. pneumophila* populations, as well as help selecting strains for further whole genome sequencing.

The principal objective of this study was to assess the current distribution of *L. pneumophila* populations from DWDS of hospitals throughout the West Bank and determine their clonal structure and genetic diversity. To this end, *Legionella* abundances were determined for two years in bulk water and biofilms in the hospitals by applying both cultivation-dependent and -independent analyses. By the cultivation analyses, 180 *L. pneumophila* isolates were obtained from water and biofilm. These isolates were subjected to MLVA using 13 loci to reveal their clonal structure and genetic diversity. In comparison to MLVA databases, the uniqueness of the MLVA-genotypes of the West Bank could be assessed. For the West Bank, the clonal structure of *L. pneumophila* was related to the different locations and habitats and to situ-SBT analysis from pneumonia patients. 

## 2. Materials and Methods

### 2.1. Study Sites, Water and Biofilm Sampling 

Drinking water in the West Bank is derived from groundwater, mainly well water, with some being provided by springs. Water was provided to most of the sampled sites by the Palestinian Water Authority, except for Ramallah (sampling site D), with Mekorot as the provider. Except for site D, water treatment consisted of chlorination in storage sites before provision to the end user. All hospitals had drinking water reservoirs for water storage.

Water samples and biofilm swabs were sampled six times during the period from October 2012 to December 2014 from eight hospitals across the West Bank (Figure S1). The hospitals had the following coordinates: hospital A (coordinates: 32° 27’ N, 35° 17’ E), hospital B (32° 13’ N, 35° 14’ E) and hospital C (32° 13’ N, 35° 15’ E) in northern West Bank, hospital D (31° 53’ N, 35° 12’ E) and hospital E (31° 46’ N, 35° 14’ E) in central West Bank, and hospital F (31° 42’ N, 35° 11’ E), hospital G (31° 33’ N, 35° 4’ E) and hospital H (31° 31’ N, 35° 5’ E) in southern West Bank. Also, samples were taken from Al-Quds University (AQU) main campus, Abu Dies, East Jerusalem (31° 45’ 18.07’’ N, 35° 15’ 37.614’’ E)**.** The six samplings twice covered the main seasons, i.e., spring (March–May), summer (June–August), and autumn (October–December). It should be noted that site D could only be sampled once for spring, summer and autumn, while all other sites were sampled twice for these seasons [30]. 

Cold and hot water (if available) was collected from a faucet close to the hospital’s drinking water reservoir and biofilm swabs were taken from faucets, showerheads, and hoses. This study is representative for Jenin, Nablus, Ramallah, Jerusalem, Bethlehem, and Hebron, going from north to south in the West Bank. The temperature, chlorine, pH, hardness, and conductivity of the water samples were determined upon collection using probes and quantofix sticks (Macherey-Nagel GmbH, Düren, Germany). Further details on sampling and individual results of the physico-chemical parameters are given in Zayed [30].

### 2.2. Cultivation-Dependent Analysis 

A total of 72 water samples were collected in sterile 1L plastic bottles after a brief flow time (2–3 min). One liter each of cold and hot water was collected for Heterotrophic Plate Counts (HPC) and again for *Legionella* counts from the hospitals. To neutralize residual free chlorine, 0.5 mL of 0.1 N sodium thiosulphate was added to the sterile bottles for *Legionella* plate counts [31]. 

For HPC, yeast agar plates (Ant. Er. CP63.1, Carl Roth, Karlsruhe, Germany) were used according to the manufacturer’s instruction for each type of water in two sets of triplicates. First, 0.1 mL of the water sample was spread on each agar plate using a sterile glass spreader. The plates were inverted and incubated; three plates each were incubated at 37 °C for 48 h and at 25 °C for 72 h.

Concerning *Legionella* plate counts, 100 mL of water sample was filtered onto a membrane filter (membrane solutions, pore size 0.45 μm, diameter 47 mm, Whatman, England) using sterile filtration unit (Nalgene, Germany). A vacuum of 200 mbar was applied. After filtration, 30 mL of acid buffer (3.9 mL of 0.2 mol/L HCl and 25 mL of sterile 0.2 mol/L KCl were mixed, pH 2.2 ± 0.2) was placed on top of the membrane filter and left for 5 min. The filter was rinsed with 20 mL Page’s saline (1.20 g NaCl, 0.04 g MgSO_4_·7H_2_O, 0.04 g CaCl_2_·2H_2_O), and 1.42 g Na_2_HPO_4_ and 1.36 g KH_2_PO_4_ were dissolved in ten liters of distilled water and autoclaved. The membrane filter was removed from the filtration unit with sterile forceps and placed onto the relevant agar plate. Duplicates of BCYE and GVPC (M809, Himedia, India) agar plates were used according to the manufacturer’s instruction. The plates were incubated inverted at 37 °C for 10 days. Plates were checked for growth twice (after three and ten days). Final counts of the triplicates were done after ten days with descriptions of the colonies.

Also, a total of 1136 biofilm swabs from the anterior surfaces of faucets, showerheads or shower hoses in all hospital wards, mainly in areas occupied by high-risk patients (intensive care unit, operating theater, oncology and surgery wards), was obtained using transport medium (Copan, Culture swab transport system, Italy). Swabs for *Legionella* identification were processed immediately by culturing on GVPC agar (medium M809, Himedia, India) based on ISO 11731:2004 [17].

### 2.3. Cultivation-Independent Analysis (16S rDNA PCR)

A total of 72 samples (five liters) each of cold and hot water was collected from the main water source from each site for DNA extraction. Water samples were filtered onto sandwich membrane filters composed of nucleopore-filter (Nuclepore Track-Etch Membrane, MB 90 mm, 0.2 µm, Whatman, UK) and glass fiber-microfilter (GF/F) (GFF, 90 mm, Whatman, UK). Also, a total of 225 biofilm swabs from the anterior surfaces of faucets, showerheads or shower hoses was obtained for DNA extraction using sterile cotton swabs (Cotton Tipped Applicator, Beijing, China).

For the extraction of DNA from the filter sandwiches and the swabs, a modified DNeasy protocol (Qiagen kit No. 69506, Hilden, Germany) was used. Briefly, sandwich filters were cut into small pieces and incubated with enzymatic lysis buffer (20 mM Tris-HCl, 2 mM EDTA, 1.2% Triton X-100 [pH 8.0]) containing 10 mg/mL lysozyme for 60 min in a 37 °C water bath. After the addition of AL buffer from the kit, the samples were incubated at 78 °C in a shaking water bath for 20 min. After filtration through a cell strainer, i.e., 100 μm (DB falcon 352360, Corning, Glendale, AZ, USA), absolute ethanol was added to the filtrate (ratio of filtrate to ethanol is [2:1]) and the mixture was applied to the spin column of the kit. After this step, the protocol was followed according to the manufacturer’s instructions. 

Three different PCRs were carried out as follows: (i) for the detection of any bacteria, the bacterial common 16S rRNA gene primers (Com), (ii) for *Legionella* genus-specific primers (Lgsp) and (iii) for *L. pneumophila* species-specific primers (Lp1) were applied [32]. Each PCR reaction was carried out using 3 µL (1 ng/µL) of DNA template in a final volume of 25 µL. Amplification was achieved using PCR-ready Master Mix (GoTaq, Green Master Mix, Promega, Madison, WI, USA).

To test the specificity of *L. pneumophila* primers and confirm species identity, six isolates were identified by amplifying and sequencing an internal fragment of the 16S rRNA gene according to Senderovich et al. [33]. The obtained sequences were compared using the NCBI service to certain closest relatives. The sequences were submitted to the GeneBank database (KX778102-KX778107). Sequencing of the 16S rRNA gene of the six isolates confirmed the presence of *L. pneumophila* (≥99.8% 16S rRNA gene similarities). 

### 2.4. Sero-Grouping of Legionella Isolates 

The serogroups of the 180 *L. pneumophila* isolates were identified by an agglutination test using *Legionella* Latex (Oxoid DR0800, Basingstoke, UK). Using this test, the isolates were sero-grouped as Sg1 and Sg 2–14. Moreover, 47 isolates were sent to the National Reference laboratory for *Legionella* infections in Dresden for analysis by monoclonal antibody subgrouping [34].

### 2.5. Genotyping of L. pneumophila Isolates

For molecular typing of *L. pneumophila* at the strain level, MLVA-13 (MLVA-12 plus 1 additional locus from MLVA-8) designated as (MLVA-8(12)) analysis was performed for 180 isolates. DNA extraction was done either directly from living biomass using (Qiagen kit No. 69504, Hilden, Germany) according to the manufacturer protocol, or from biomass on FTA cards (Whatman, Sigma-Aldrich, Darmstadt, Germany). 

For DNA extraction from the FTA cards, the area of the card containing the biomass was punched into 3 mm circular pieces. The pieces were transferred to 0.5 mL sterile water (Roth, Karlsruhe, Germany), incubated for 3 min at room temperature and vortexed three times (after water addition, after 1 min and after 3 min incubation). The FTA punch was removed and 1× Tris-EDTA buffer (Sigma-Aldrich, Darmstadt, Germany) was added to the water to preserve the DNA from degradation. More details on the FTA technology are given by Rajendram et al. [35]. DNA was finally quantified by Nanodrop spectrophotometer (NanoDrop, Thermo Scientific, Dreieich, Germany). MLVA-8 and MLVA-12 molecular genotyping assays by multiplex PCR and capillary electrophoresis were carried out for all isolates, as detailed by Pourcel et al., Sobral el al., Visca et al. and Pecellin [28,29,36,37]. 

For comparison, a subset of strains representing all MLVA-genotypes was characterized by sequence-based typing (SBT) [38]. In addition, several *L. pneumophila* (Lpn) reference strains were used to generate MLVA-8(12) profiles for comparison and interpretation of the results; these reference strains were Lpn str. Philadelphila-1 (ATCC 33152^T^) [39], Lpn str. Paris (CIP 107629) [40], Lpn str. Bloomington-2 (ATCC 3315) and Lpn str. Corby (NC_009494) [41]. 

### 2.6. Statistical Analysis

Statistical analysis was performed using the GraphPad Prism software v7.0 (Graph-Pad, San Diego California, USA), and cluster analysis and a phylogenetic tree were constructed using PRIMER software v7.0.7 (Primer-e, Auckland, New Zealand). Non-normalized data were normalized. Data are presented as means ± standard deviation (SD). An agglomerative clustering dendrogram was created using the PRIMER software in order to study the similarities between the genotyping characteristics of *L. pneumophila* strains belonging to different VNTR markers (Lpms). The resemblance matrix was calculated using the Bray-Curtis index of association on the VNTR marker.

Capillary electrophoresis data analysis and calculation of the number of repeats for each VNTR marker were performed as described in Pourcel et al. [28]. The numerical code used to designate the MLVA-8 and MLVA-12 genotypes, as well as the joint code for the MLVA-8(12) genotypes, were continued for the isolates. Null alleles (“0”) were assigned when no amplicon was detected. Cluster analysis was performed in Bionumerics (version 5.0, Applied Maths, Gent, Belgium). The UPGMA (Unweighted Pair Group Method with Arithmetic Mean) method using a categorical coefficient was applied to define the clusters. The MLVA-8 profiles obtained in this study were compared to those from the *Legionella* MLVA-database, and clusters were defined applying a cut-off of 60% similarity, as done previously [29]. Minimum spanning trees were performed using the categorical coefficient. Simpson’s Index of Diversity coefficient was calculated using the online tool provided in https://www.easycalculation.com/statistics/simpson-diversity-index.php. To measure the variation of the number of repeats at each VNTR locus, the Hunter-Gaston Discrimination Index (HGDI), which is a modification of the Simpson’s Index of Diversity, was calculated according to Pecellin [37].

## 3. Results

### 3.1. Biological and Physico-chemical Characteristics of Drinking Water Distribution Systems (DWDS)

The sampled drinking water of the eight hospitals was mainly groundwater based and characterized by a high hardness (on average 230–300 mg/L CaCO_3_ equivalents) and high conductivity (on average 650–900 µS) (Table 1). The average temperature of the cold water ranged between 21.1 °C and 24.3 °C. The average temperature of hot water ranged between 38.6 °C and 51.9 °C. The average pH of the cold and hot water was 7.6 and 8.0. The conductivity of the hot water was higher than that of the cold water in all hospitals. Chlorine varied on average between 0.2 and 0.7 mg/L. Heterotrophic plate counts at 37 °C ranged from 1.7 × 10^4^ to 1.6 × 10^5^ CFU/L. 

During the study period, *L. pneumophila* was detected in the DWDS of all hospitals and at Al-Quds University. The sampling comprised six campaigns, twice covering the main seasonal changes. Sampling comprised water and biofilms, with comparable numbers of samples taken from each hospital.

From water samples, five *L. pneumophila* strains were isolated from 72 samples. *L, pneumophila* was isolated only from the drinking water of three hospitals (A, F and G). For the collection period, the *Legionella* counts per hospital ranged from 0 to 150 CFU/L (Table 1). In hospital A, on average, 43 CFU/L of *Legionella* spp from cold water were detected; in hospital F, 150 CFU/L of *Legionella* spp were detected from cold water and 91 CFU/L from hot water; finally, in hospital G, on average, 8 CFU/L *Legionella* spp were detected from cold water. Hot water was not continuously available from all hospitals, but *Legionella* spp counts were comparable to cold water [42]. *Legionella* spp, mainly *L. pneumophila,* was isolated from 191 out of 1136 biofilm swab samples (16.8%) (Table 2). The majority of *Legionella* positive samples were detected in 2012 (23.5%), while the fewest samples tested positive in 2013 (8.7%). The highest frequency of *L. pneumophila* in biofilm swabs was detected in hospital F (26.3%), where *Legionella* spp were also detected in the DWDS during 2012–2014. Meanwhile, the lowest frequency was detected in hospital C (3.3%). Finally, a high frequency of *L. pneumophila* was detected in Al-Quds University (36.4%) during the only collection in 2012. 

### 3.2. Cultivation Dependent Analysis (CDA) Versus Cultivation Independent Analysis (CIA)

Although CDA is the standard and recommended technique for environmental surveillance of *L. pneumophila,* CIA provides higher sensitivity and overcomes the problems of CDA for *Legionella* because of the VBNC state and its overgrowth by competing bacteria. In this study, both methods were used to detect *Legionella* spp in hospital DWDS (Table 2).

A total of 72 water samples and 225 biofilm swabs from the eight hospitals were tested by conventional PCR using three different primers (com, Lgsp, Lpn). Almost all of the samples were positive using com primers (n = 71, 98.6% and n = 225, 100%) for water samples and biofilm swabs respectively. *Legionella* spp were detected in biofilm swabs more than in water samples (n = 167, 74.2% and n = 42, 58.3%), respectively (Table 2). Similar results were obtained using *L. pneumophila*-specific primers: 60% of the biofilm swabs and 50% of the water samples were positive. As expected, the PCR-based CIA showed higher sensitivity than CDA. CIA analysis increased the detection of *L. pneumophila* from 8.3% (CDA) to 50% (CIA) for water samples, and from 16.8% (CDA) to 59.5% (CIA) for biofilm samples. 

### 3.3. MLVA-8(12) Genotypes of L. pneumophila Isolates

MLVA genotyping was carried out for the 180 isolates. MLVA-8, as well as MLVA-12 and the joint scheme MLVA-8(12), were analyzed for the study of the population of *L. pneumophila* isolates. The 180 isolates were categorized as 16 MLVA-8 genotypes (Index of Diversity ID = 0.771, 95% Confidence Interval CI 0.721–0.822), 25 MLVA-12 genotypes (ID = 0.790, 95% CI, 0.739–0.841) and 27 MLVA-8(12) genotypes (ID = 0.790, 95% CI 0.739–0.841). This indicates a lower genotypic resolution for MLVA-8 using eight loci compared to MLVA-8(12) using a total of 13 loci. For details on the comparison based on a larger set of *L. pneumophila* isolates, see [37].

**Figure 1:**
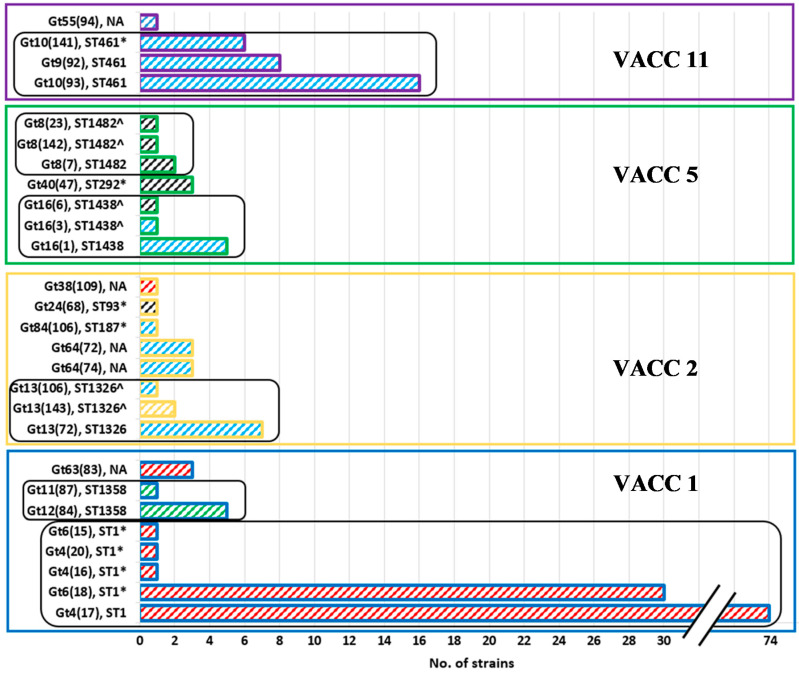
Comparison of the number of strains per MLVA8(12)-Genotype (Gt) of the isolated 180 *L. pneumophila* strains and their respective sequence types (ST). Each group of bars, outline color and colored frame indicate VACCs as follows: blue outline represents VACC1, yellow outline represents VACC2, green outline represents VACC5 and purple outline represents VACC11. The black round-edged frames indicate the group of genotypes from the same ST. The wide upward diagonal hatches inside the bars indicate Sg as follows: Sg1—red, Sg 6—sky blue, Sg8—green, Sg10—yellow and Sg2 to Sg14—black. NA—not available ST; *, ST was assessed for strains of the same MLVA-8(12) genotype, and not directly for the West Bank strains; ^^^, ST was estimated from the MLVA-8 pattern.

The use of the MLVA-8(12)-genotype nomenclature made it possible to directly compare strains genotyped from MLVA-8 and MLVA-12: the first number reflects the MLVA-8-classification, while the number in brackets reflects the 12 loci-classification, e.g., Gt 4(17) is a Gt 4 according to the MLVA-8, and a Gt 17 according to MLVA-12. Fourteen MLVA-8(12) genotypes were represented by 2 to 74 strains, whereas 13 genotypes were represented by just a single strain from the West Bank isolates (Figure 1). The MLVA-8(12) genotypes comprising the most strains were Gt 4(17), Gt 6(18) and Gt 10(93).

### 3.4. VNTR Clonal Complexes (VACC) and Relationship among the Genotypes 

**Figure 2:**
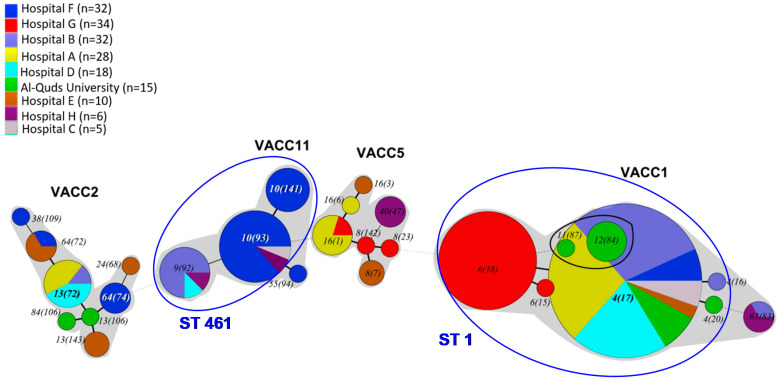
Minimum-spanning tree based on MLVA-8(12) profiles of 180 *L. pneumophila* strains isolated from the West Bank. Each circle in the tree represents a different MLVA-8(12) genotype. The genotype number is indicated within or near the circle, whose size is proportional to genotype frequency. Different colors in the pie charts refer to the eight sampling locations (see legend). The thickness of the branches represents the number of different loci. MLVA clonal complexes (VACC) are shaded in grey. The circles representing the Sg8-singletons from Al-Quds University, i.e., Gt 11(87) and Gt 12(84), overlap visually within the circle that represents Gt4(17) due to the high abundance of this genotype. Blue ellipses indicate genotypes that could be candidates for LD. These genotypes belong to ST1 and ST461 (indicated in blue letters) and were assessed by in situ-SBT and present in half of the LD cases in a West Bank study [43].

An analysis of the relationship among the genotypes was achieved by UPGMA-based cluster analysis of the MLVA-8(12) profiles of the 180 *L. pneumophila* strains (Figure S2). The MLVA clonal complexes (VACC) were defined by a cutoff level of 60% similarity. In addition, the genetic relationship among genotypes was estimated by a minimum-spanning tree based on the MLVA-8(12) profiles (Figure 2).

All MLVA8(12) genotypes were clustered into four MLVA clonal complexes or VACCs (VACC1, VACC2, VACC5 and VACC11) (Figure 2). VACC1, VACC2 and VACC5 were clonal complexes previously defined in the MLVA *Legionella* database. VACC11 is described for the first time in this study (Figure 2 and Figure S2). VACC1 was the largest cluster, including 110 isolates (61.6%). VACC11, VACC2 and VACC5 were, in comparison, smaller clusters, comprising 31, 19 and 14 isolates, respectively (Figure 1).

Not all strains could be included in VACCs. A small group of six isolates that belonged to two different genotypes, i.e., Gt 11(87) and Gt 12(84), separated from the large VACC1 and remained as singletons, i.e., they could not be directly included in a VACC. They differed from the rest of the isolates contained in VACC1 in the number of repeats observed for VNTR markers Lpms31, i.e., 17, in comparison to 4 or 0 in the rest of the profiles of VACC1, and VNTR Lpms33 and Lpms34, which both presented only one repeat in contrast to the 4 and 2 repeats found, respectively, in VACC1. In total, 96.6% of isolates (n = 174) were clustered into the four VACCs, and only 3.6% (n = 6) were found as singletons. 

**Figure 3:**
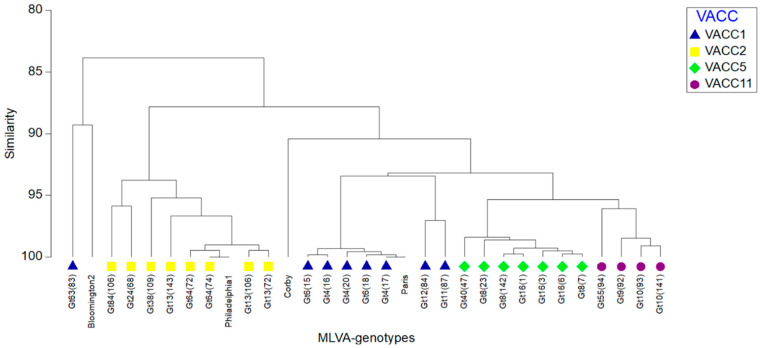
Single-linkage cluster dendrogram representing the percentage of similarity between MLVA-8(12) profiles of the genotypes retrieved from the West Bank. For a comparison, MLVA-8(12) profiles of *L. pneumophila* reference strains (Bloomington2, Philadelphia-1, Corby, Paris) were added. The resemblance matrix was calculated using the Bray-Curtis index of association. Strains of different VNTR clonal complexes (VACC) are indicated in different colors (see insert).

In addition, single linkage clustering using the Bray-Curtis-Index of association was performed with a cutoff value of 60% corresponding to 13 VNTRs. The MLVA8(12) profiles of four additional reference strains were added to the dataset to show their relationship with the genotypes in this study (Figure 3). This clustering showed the same four groups corresponding to the VACCs observed with the UPGMA-clustering and the minimum-spanning tree (Figure 2 and Figure S2). In addition, it revealed a very close relationship of the *L. pneumophila* strain Paris with Gt 4(17) in VACC1, and of Gt 64(74) with reference strain *L. pneumophila* strain Philadelphia1 in VACC2. The dendrogram showed a high discriminatory power and subclustering between *L. pneumophila* isolates with similarity of (>95%). Gt63(83) was shown as an outlier due to the three “Null” alleles in Lpms31, Lpms34 and Lpms35, i.e., no PCR product was obtained for these VNTR-markers (Figure 3). Overall, the Bray-Curtis-grouping of the genotypes confirmed the four VACC cluster. Also, the aforementioned singleton genotypes Gt11(87) and Gt12(84) were grouped separately from VACC1.

Overall, a more detailed analysis of the population structure at the level of the 13 VNTR markers showed a balanced variability in the number of repeats for most VNTR markers among the isolates (Table 3). This could be due to the homogenized habitat and location where the isolates were obtained. Some VNTR markers appeared to be less variable and showed a reduced number of repeats, e.g., Lpms3, Lpms17 and Lpms19, while others showed a greater variability, e.g., Lpms31 and Lpms35. In general, the same repeats prevailed independently of the area from which the isolates had been isolated. Null alleles were present at different frequencies in distinct VNTR markers. Especially high were the frequencies of null alleles in Lpms38. Remarkably, a new allele of Lpms34 was described during this study. The new allele had a size of 634 base pairs and was formed by four repeats. A total of 31 isolates (17.2%) contained this allele. This allele has not previously been described in MLVA studies for *L. pneumophila*.

### 3.5. Diversity within the Clonal Complexes (VACC)

The minimum-spanning tree (Figure 2) make it possible to provide an overview of the diversity and the genetic relationship among the MLVA genotypes. While the grouping by VACCs gives a first estimate of the relationship among the total of the genotypes, the relationship of the genotypes within the VACCs is also indicative. 

In VACC1, all genotypes pertaining to ST1 were closely related to Gt 4(17). The replicate number of only one locus had changed compared to Gt 4(17). This is comparable to VACC11, where Gt 10(93) has this central position, with only one locus being different compared to Gt 10(141), Gt 9(92) and Gt 55(94). By contrast, in VACC2 and VACC5, a set of more distantly related strains with a broader set of different changes in the VNTR-loci was observed. 

The relationship of the genotypes within the cluster as reflected by the minimum spanning tree were consistent with the results of the UPGMA-based cluster analysis (Figure S2) and Bray-Curtis-based analysis (Figure 3). 

### 3.6. Comparison of MLVA-8(12) Genotypes and Clonal Complexes with Sequence Types (ST)

In our comparison, 22 of 27 MLVA8(12) genotypes could be assigned to nine sequence types (STs). Most STs with a larger set of strains could be divided in two to five genotypes (Figure 1 and Figure S2). ST1 was split into five MLVA-8(12) genotypes all adhering to VACC1. ST1 comprised the largest fraction of strains, i.e., 111 strains of the total of 180 strains. ST1 comprised the MLVA-8(12) genotypes with the most isolates, i.e., Gt 4(17) and Gt 6(18) (Figure 1 and Figure 2). ST461 comprised 30 strains and three genotypes of VACC11. It was the second largest ST. VACC11 strains were not present in any other ST. Three genotypes were also present in each of ST1326, ST1438, and ST1482. Two MLVA genotypes were present in ST1358. The remaining three STs (9, 93, 187) comprised one to three strains and constituted a single MLVA-8(12) genotype. 

In summary, all STs with a larger set of strains were split up in several MLVA-genotypes, i.e., MLVA-8(12)-genotyping showed a substantially higher resolution than SBT. All STs comprised only strains of the same VNTR clonal complex (VACC). Our dataset indicates a high level of consistency between SBT and MLVA8(12)-genotyping. This observation was confirmed by the analysis of a larger and more diverse set of *L. pneumophila* isolates [37]. 

### 3.7. Serotype Distribution of L. pneumophila Isolates and Relationship with MLVA Genotypes and Clonal Complexes

The 180 PCR-confirmed *L. pneumophila* environmental isolates were tested for serogroups (Table 4 and Figure 1). Most of the isolates were characterized as serogroup 1 (Sg1) (n = 111); the remaining 69 isolates were non Sg1. A subset of ten Sg1 isolates was subgrouped according to the monoclonal antibody; all belonged to the MAb 3/1 negative OLDA subtype, which is considered to lack the virulence- associated epitope. The 69 non-Sg1 isolates were analyzed by monoclonal subgrouping; 54 of them were serotyped as Sg6, followed by Sg8 (n = 6) and Sg10 (n = 2). The rest of the non-Sg.1 were characterized as serogroups 2–14, as determined using an agglutination kit.

In terms of the number of isolates, the *L. pneumophila* population showed a dominance of Sg1, followed by Sg6 (Figure 1). In terms of MLVA-genotypes, 11 could be attributed to Sg6 and seven to Sg1, while Sg8 was represented by only two and Sg10 by only one genotype. In terms of clonal complexes, VACC11 comprised only strains of Sg6. VACC1 comprised mainly Sg1 strains, except for Sg8 for genotypes Gt11(87) and Gt12(84). VACC5 comprised strains of either Sg6 or Sg2–14. By contrast, VACC2 comprised a serogroup-divers set of genotypes, i.e., Sg1, Sg6, Sg10 and Sg2–14. The six singleton isolates were all Sg8-strains isolated from Al-Quds University. 

### 3.8. Prevalence and Abundance of L. pneumophila MLVA-genotypes and Clonal Complexes (VACCs) 

An overview of the association of the MLVA-8(12) genotypes and VACCs with the different sampling sites is reflected in the minimum-spanning tree (Figure 2). Details on the strains retrieved and their characteristics for each sampling site are listed in Table 5.

In terms of VACC prevalence, Figure 2 shows that at least two distinct clonal complexes were present at each hospital as well as at the Al-Quds University. VACC1, the largest clonal complex, was present across the West Bank. Genotypes belonging to it were isolated from all eight hospitals and from AQU. VACC2 isolates were isolated from four hospitals distributed throughout the West Bank and AQU. Although VACC11 was present at five hospitals, it was the major clonal complex at hospital F (n = 22, 66.7%). Isolates grouped into VACC5, the smallest clonal complex, were, however, found at four hospitals located throughout the West Bank (Table 5).

At the genotype level, only nine out of the 27 MLVA-8(12) genotypes were isolated in more than one location. The remaining 18 MLVA-8(12) genotypes were isolated exclusively in one specific site. Gt 4(17), the main VACC1-genotype that also comprises *L. pneumophila* strain Paris, was the only genotype present in all hospitals except for hospital G. Furthermore, it represented a high fraction of the isolates in several hospitals. Gt 4(17) was the most abundant genotype in hospital B (68.7%), A (71.4%), D (83.4%), and C (80%). At Al-Quds University, Gt 4(17) accounted for 40% of the isolates. Gt 6(18) was the second most abundant VACC1-genotype; it is closely related to GT 4(17), differing by just one additional repeat in the VNTR Lpms35. Gt 6(18) was endemic in the West Bank and found exclusively in hospital G, where it was the most abundant genotype (90.9%). Genotype Gt 10(93), a member of the newly-described VACC11, was found in hospitals C and F; it was isolated only once in hospital C, but was the most abundant genotype in hospital F (n = 15, 45.5%). The remaining genotypes that were found in more than one location had a rather restricted distribution, i.e., they were observed only in one or two additional sampling sites (Figure 2 and Table 5).

In general, the most frequent genotypes in each hospital were isolated repeatedly during samplings performed in following years. Gt 4(17) was recurrently isolated in hospitals A, B, C, D and F between 2012 and 2014. The endemic Gt 6(18) was isolated in hospital G in 2013 and 2014. Genotypes Gt 10(141), Gt 10(93), and Gt 9(92) were isolated in their respective sites (hospital F, B) from 2012 to 2014. The MLVA-8(12) genotypes were shared among the north, central and southern West Bank. According to the geographical distribution of the West Bank, 64 (35.6%), 43 (23.9%) and 73 (40.6%) isolates were isolated from northern, central and southern areas, respectively. Nevertheless, the most abundant and broadly distributed genotype was common to the whole West Bank, i.e., Gt 4(17), which comprised 68.8%, 53.5% and 9.6% of total isolates in northern, central and southern West Bank, respectively. Surprisingly, four genotypes, i.e., Gt 9(92), Gt 10(93), Gt 16(1) and Gt 63(83), were shared between more distant sites, i.e., northern and southern West Bank. As a tendency, the diversity of the genotypes observed decreased from the Southern to the Northern West Bank. 

Interestingly, Gt 4(17) was never obtained from water samples, but only from biofilm. Genotypes of rather limited distribution were the only genotypes retrieved from water, i.e., Gt 10(93) and the endemic Gt 10(141), were obtained from hospital F, the endemic Gt 6(18) from G, and Gt 16(1) from A, respectively. 

In summary, two thirds of the MLVA-8(12) genotypes were endemic, i.e., they were found exclusively in one hospital or the Al-Quds University. Only one third of the MLVA-8(12) genotypes were isolated in more than a single location, and these common genotypes were usually much more frequent in one of the locations. An exception was Gt 4(17), that was present in most locations and occurred often in high abundance. 

## 4. Discussion 

### 4.1. Legionella Abundance in Hospital Water and Biofilm of the West Bank

This study is based on the first extensive sampling campaign examining the prevalence of *Legionella* spp in DWDS of hospitals in the West Bank. The analysis of water and biofilm samples was done using cultivation-dependent and -independent methods targeting *Legionella* from the genus to the clone level for *L. pneumophila* by molecular techniques including MLVA-8(12) genotyping.

In general, water samples had a far lower prevalence of *Legionella* compared to biofilms. Water samples tested positive for the presence of *Legionella* with a prevalence of 8.3% by cultivation dependent analysis and 50% by cultivation independent analysis. Biofilms had a higher prevalence, with 16.8% positive by cultivation dependent analysis and 61.3% by cultivation independent analysis. The findings of increased PCR-based detection in water and biofilms are consistent with other studies [44] and were analyzed in detail by Zayed et al. [42].

The MLVA-genotypes of the five water isolates were always present in the biofilm of the respective sites, usually making up a high fraction of the local biofilm isolates. In more detail, only from hospital A (one isolate), hospital F (three isolates) and hospital G (one isolate) were isolates from water obtained. In hospital A, the most abundant genotype from biofilm was Gt 4(17) (71% of the isolates), whereas Gt 16(1) obtained from water had a lower abundance (11%) of the biofilm isolates of this site. The three water isolates from hospital F belonged to Gt 10(93) or Gt 10(141). The water isolate from hospital G belonged to Gt 6(18). The water isolates from hospital F and G were the most abundant biofilm genotypes from these hospitals, i.e., Gt 10(93), Gt 10(141) and Gt 6(18) (Table 5 and Table S1). 

The low prevalence of *Legionella* in culture-based studies is in accordance with studies in Israel and Greece [45,46,47,48]. However, many studies showed a much higher culturable *L. pneumophila* prevalence in water, e.g., 21.6%, 22% and 40% in Kuwait, Tunisia [49,50] and Jordan [51], respectively. The prevalence of *L. pneumophila* was even higher (68.5%) in a study from northern Israel [52]. The low prevalence of *L. pneumophila* in the West Bank was, at least to some extent, attributed to the high magnesium content of the drinking water [42]. 

Most of the *L. pneumophila* isolates from the West Bank (n = 175, 97%) were obtained from biofilm samples (Table 2). This is consistent with the results by Douterelo et al. [53], showing that more than 95% of the microbial biomass in a DWDS is found in the biofilms attached to the pipe lines due to the multiple advantages that biofilms represent for microorganisms, such as providing protection from external factors and beneficial interactions with other microorganisms [54]. Additionally, from the point of view of public health, biofilm sampling has a great importance, since it has been observed that *L. pneumophila* strains derived from biofilm replicate significantly more in murine macrophages than plankton-derived strains [55].

### 4.2. General Health Relevant Aspects of the Isolated Strains

Cultivation is still considered the gold standard for the detection of *Legionella* in the environment, even though other, nonculture methods are available, such as serology or nucleic acid-based detection methods [56]. Cultivation can be inaccurate as a result of overgrowth by other microorganisms on the agar plates, and can be ineffective due to the presence of viable but nonculturable (VNBC) *Legionella* cells [57]. However, cultivation makes it possible to obtain isolates that can be identified and characterized phenotypically and genetically, which is essential for epidemiological studies.

According to current epidemiological data available from around the world, different *L. pneumophila* serogroups cause legionellosis with a distinct estimated risk. Overall, the great majority of strains isolated from the area under study were characterized as Sg1 (62.3%). This fact followed the tendency already reported by other studies that have described Sg1 as the most frequently detected Sg of environmental isolates in different geographic regions [58,59,60]. Besides the high prevalence of Sg1, other serogroups were isolated, where the fraction of non-Sg1 isolates went up to 37.7%. In our study, Sg6 was particularly abundant (30%). Sg8 and Sg10 were also isolated, although in smaller proportions (3.3% and 1.1%, respectively) (Table 3). The results obtained here were consistent with those of two studies on the distribution of *L. pneumophila* serogroups not related to human disease in man-made water systems [60,61], and were comparable, climate-wise to the area of study, i.e., Greece [48,62] *L. pneumophila* Sg1 was the most frequently isolated serogroup, followed by Sg6 in France and the UK, where Sg10 was also found. Sg6 is the serogroup which is second most responsible for cases of LD after Sg1, according to European surveillance data [63]. Furthermore, these specific serogroups (Sg1 and Sg6) are the most frequent and virulent among clinical cases [34,64,65,66]. 

ST1 was the most prevalent sequence type in the West Bank, and is the most dominant ST worldwide [22,60,67,68]. The high abundance of ST1 in the environment has been reported in several studies. In Japan, as well as in South Korea, the majority of environmental isolates comprised ST1 [69]; for the latter, ST1 was distributed across all sampled facilities and regions and accounted for 48.1% of the isolates [70]. ST1 was the most abundant sequence type among environmental isolates in Canada, and was found ubiquitously across the country [22]. In a study conducted across the United States, ST1 was the most frequent sequence type between both clinical sporadic and environmental isolates, accounting for 25% and 49% of the total number of isolates, respectively [68]. In Europe, ST1 has also been reported as the most predominant sequence type among environmental isolates in Germany [71], England and Wales [60], Portugal [72], Spain [73], France [74] and Italy [75]. 

Typically, the climate in the West Bank is Mediterranean, slightly cool to cold in winter and dry to humid and warm to hot in summer. Previous studies have suggested that the incidence of LD may increase under warm and wet meteorological conditions, which could be exacerbated by global climate change [76]. Therefore, surveillance of environmental sources and proper maintenance of man-made freshwater systems is key in the prevention of legionellosis. Surveillance of *Legionella* in the environment is also essential to validate the efficacy of decontamination procedures, and for risk assessment when evaluating potential transmission or amplification sources. 

### 4.3. Genotyping Using MLVA—What Resolution Is Needed for Ecological and Clinical Issues?

SBT is considered as the gold-standard for *L. pneumophila* genotyping, primarily due to a large International database created by the “*L. pneumophila* community”. SBT has high typeability, interlaboratory reproducibility and generally a high index of discrimination [77]. However, the resolution is not as high as is often needed, e.g., for ST1 and its many health-relevant strains occurring world-wide [78,79]. It is suggested that the number of sequenced genes be increased to about fifty, guided by genome analyses. 

MLVA is a rather well-established genotyping method currently used for 32 pathogenic bacterial species [27]. To date, it has been applied mostly for clinical strains. MLVA-8(12) for *L. pneumophila* was developed and its resolution analyzed by Sobral et al. Visca et al. and Pecellin [29,36,37]. All these studies demonstrated that MLVA-8, and even more, MLVA-8(12), have a higher resolution than SBT and are rather consistent with SBT. This was also shown in this study: larger strain sets adhering to a specific ST could always be distinguished into different MLVA-genotypes. MLVA-genotyping was always consistent with SBT, i.e., strains of the same genotype were not assigned to different STs. In this study, nine STs were split into 22 MLVA-8(12) genotypes, with large STs comprising several MLVA-genotypes.

The required resolution for the genotyping of strains is dependent on the tasks to be performed. For clinical reasons, genotyping should allow for a distinction with respect to virulence traits and antibiotic resistance [80]. Sharaby et al. [81,82] showed that *L. pneumophila* isolates from Israel had MLVA-8 genotype-specific virulence traits. Even their resistance to antibiotics showed a strong correlation with the MLVA-genotypes. Interestingly, environmental strains were more resistant to antibiotics than clinical ones. Moreover, there were major differences between different MLVA-genotypes associated with ST1 that could not have been distinguished by SBT.

For the management of *L. pneumophila* abundance in DWDS, the ecology of *L. pneumophila* has to be studied. As first shown by Rodriguez-Martinez et al. [52], the MLVA-genotyping level makes it possible to distinguish among specific ecotypes, i.e., by assessing the strains’ environmental preferences. It was demonstrated that the preference for temperature and the respective growth speed could be well differentiated using MLVA-genotyping [83]. Furthermore, in these studies, there were relevant differences between different MLVA-genotypes affiliated with ST1. A more detailed analysis on environmental preferences for the West Bank strains was provided by Zayed et al. [42]. For the West Bank strains, specific environmental traits could be assigned to all MLVA-8(12) genotypes comprising a larger set of strains. 

Microbial source tracking is of high relevance for *L. pneumophila* due to its occurrence in environmental freshwater and its transfer, specifically during outbreaks, from the environment to humans. MLVA is rather economical and can be run in a fully automated manner using capillary sequencing. Due to the need for rapid analyses of large sets of strains in an outbreak scenario, MLVA can be a cost-efficient and fast option. The successful source tracking by MLVA of an *L. pneumophila* outbreak was successfully demonstrated by Sobral et al. [29] in the French city of Rennes.

Another point of interest is intraspecies evolution. Garcia et al. [84] successfully followed the microevolution of *Vibrio parahaemolyticus* using MLVA technology in an experimental setting. Based on the experimentally-derived mutation rates, they estimated the worldwide evolution and time scale for *V. parahaemolyticus* populations. In general, clustering in VACCs gives the basis for good estimates among MLVA-genotypes. Links between the strains indicate their lines of evolution. For this study, relationships of the occurrence of genotypes over time and in neighboring sampling sites could indicate source strains and evolutionary tendencies [37]. 

### 4.4. Comparison of L. pneumophila MLVA-8(12) Genotypes from the West Bank with the International Data Base and A Study on A Larger Set of Strains from Germany and Israel 

To address the distribution of the 27 MLVA-8(12) genotypes outside of the West Bank, the genotypes were compared to the International MLVA database (http://microbesgenotyping.i2bc.paris-saclay.fr/) and a larger strain analysis was performed by Pecellín [37]. The study by Pecellín [37] described a set of 610 clinical and environmental *L. pneumophila* strains retrieved from Germany, Israel and Palestine. The set of 180 strains from the West Bank described in this study were included in the study by Pecellín [37].

A comparison with the international database and the study by Pecellín [37] showed that there were a few highly ubiquitous genotypes. First of all, the MLVA-genotype comprised *L. pneumophila* Paris, i.e., Gt 4(17), that was associated with VACC1 and ST1. This genotype has a high relevance worldwide as a clinical and environmental genotype [68,74]. Another prominent member is Gt64(74), that comprises *L. pneumophila* strain Philadelphia-1 (ST 440). Two more genotypes occurred in the study of the three countries, i.e., Gt 4(16), a ST1-genotype closely related to Gt 4(17), and Gt 24(68), which is related to ST93. Gt 4(17) has an eminent role in Israel and the West Bank. This genotype occurs at high abundances in both regions. In Israel, it plays a relevant role as a clinical isolate [81]. At Oranim campus close to Haifa, concentrations of Gt 4(17) were high in the water and adjacent biofilms. In comparison with Germany, one more genotype occurred, i.e., Gt 40(74), which was associated with ST292. In comparison with Israel, two genotypes of environmental and clinical origin were observed, i.e., Gt 6(18) and Gt 6(15), both of which were affiliated with ST1 [81]. 

In summary, this means that 20 of the 27 MLVA-genotypes were unique for the West Bank. In addition, the VNTR clonal cluster VACC11 was described in the present study for the first time for the West Bank. Both the high percentage of new genotypes and the new VACC indicate the uniqueness of the West Bank strains. One possible line of ecological reasoning could be that groundwater is mostly the source water for DWDS in the West Bank [85], with an individualistic supply due to specific wells and springs as local water sources. This high diversity among sampling sites and the uniqueness of most of the genotypes may therefore be due to the very diverse water sources (Figure 2 and Table 5).

### 4.5. Conceivable Health Relevance of the L. pneumophila MLVA-8(12) Genotypes in the DWDS 

PCR analysis of sputum and Broncho-Alveolar-Lavage (BAL) samples from pneumonia patients by Jaber et al. [43] in the West Bank revealed a rather high fraction of *L. pneumophila* contamination, i.e., 15% and 35%, respectively. By in situ SBT, they identified 29% of the detected *L. pneumophila* contaminations as ST1, and 21% as ST461. This is rather consistent with the MLVA-8(12) genotypes retrieved from the DWDS of the West Bank: the largest fraction of strains retrieved were affiliated with ST1 and ST461 (Figure 2). Unfortunately, there were no isolates obtained from patients due to previous antibiotic treatment. Therefore, no clinical strains could have been submitted to MLVA-8(12) analysis [43].

In the study by Sharaby et al. [81], clinical strains associated with ST1 were mostly Gt 4(17) followed by Gt 6(18). Therefore, it can be assumed that these MLVA-genotypes may have been responsible for infections in patients where ST1 was detected. For patients with ST461, all strains affiliated with this ST could be responsible for pneumonia, i.e., all genotypes (Gt10(141), Gt 10(93), Gt 9(92)) of the newly-described VACC11. The role of the remaining genotypes from the West Bank (non-ST1 and non-ST461) is unknown and remains to be elucidated in further studies. Hints may come from some publications [29] where strains of VACC2, including the genotype of *L. pneumophila* Philadelphia-1, were often observed as sources of LD in local outbreaks. 

Another relevant point for public health and infection is the concentration of *L. pneumophila* in drinking water. For a site with comparable climate, Sharaby et al. [86] showed the risk at high concentrations of culturable *L. pneumophila* in drinking water of the Oranim campus (Haifa). From this perspective, it can be regarded as good news that the level of culturable *L. pneumophila* in the West Bank hospitals was, on average, rather low or undetectable. However, the noncontinuous water supply in the West Bank may cause disruption of biofilms in the DWDS, leading to short-term increases of the levels of *L. pneumophila* in drinking water that remain undetected during measurement campaigns.

Another health aspect is DWDS in private homes that has not yet been assessed. Since *L. pneumophila* STs found in drinking water were observed for at least half of the investigated pneumonia patients, the drinking water in private homes of the West Bank should be considered as a potential health risk with respect to LD. Overall, the situations in private homes, i.e., discontinuous water supply and roof storage of water, are still underassessed; future studies should investigate the risks associated with the water quality and supply aspects. 

In summary, our study provided the first comprehensive and long-term overview of the prevalence of *L. pneumophila* in DWDS with respect to water and biofilm, achieved by cultivation and PCR-based methods. Genotyping of the isolated *L. pneumophila* strains by high-resolution genotyping methods allowed us to group the isolates on a subspecies level and make international comparisons. In combination with genotype abundance and regional distribution, this provides better insights into potential health risks and may indicate where and which prevention measures might be needed. 

## 5. Conclusions

A two-year study of *L. pneumophila* populations in water and biofilms in the drinking water distribution systems (DWDS) of eight hospitals across the West Bank demonstrated low and rare abundance of culturable *L. pneumophila* in water, but substantially higher prevalence in biofilm. PCR-based analyses consistently showed a higher detection rate in water and biofilm. Based on high resolution MLVA-8(12) genotyping, the 180 isolates retrieved in the West Bank could be characterized as a rather diverse population, with four clonal complexes (VACC). Most of the genotypes (20 out of 27) were unique, and so far, have only been described for the West Bank, including those forming a new clonal complex (VACC11). In addition, seven genotypes were also observed outside of the West Bank, including two genotypes of worldwide abundance, i.e., Gt 4(17) comprising *L. pneumophila* strain Paris, and Gt 64(74) comprising strain Philadelphia-1. The observed uniqueness of the genotypes and the variability from site to site were attributed to individual groundwater-based water supplies. In addition, the isolated strains seemed to be of high health relevance, especially strains of VACC1 and VACC11. MLVA-genotyping was shown to be highly consistent with SBT but showed a higher resolution. Since the most health relevant ST1 (VACC1) and ST461 (VACC11) strains could be further distinguished into several MLVA-genotypes, MLVA-genotyping could provide an excellent basis for future source tracking in the West Bank. MLVA-genotyping provides an adequate resolution, and thus, a good basis for detailed studies of the health- and water-management-relevant traits of *L. pneumophila* [42,81,83,86] in support of a better clinical and DWDS management in the West Bank.

## Figures and Tables

**Table 1:** Average of biological and physico-chemical parameters from the drinking water systems (DWDS) of the eight hospitals in the West Bank.

Hospital	Water Type	HPC (CFU/L) at 37 °C ± SD	HPC (CFU/L) at 22 °C ± SD	*Leg*. Count (CFU/L) ± SD	Tempe-rature °C ± SD	pH ± SD	Conductivity µS/cm ± SD	Chlorine mg/L ± SD	Hardness (mg/L)
A	Cold	2.1 × 10^4^ ± 1.6 × 10^2^	7.4 × 10^3^ ± 7.1 × 10^1^	4.3 × 10^1^ ± 1.06 × 10^2^	23.1 ± 2.4	7.7 ± 0.2	780 ± 158	0.5 ± 0.3	290 ± 36
	Hot	NA	NA	NA	NA	NA	NA	NA	NA
B	Cold	2.0 × 10^4^ ± 2.2 × 10^2^	1.4 × 10^3^ ± 1.4 × 10^2^	BD	24.3 ± 2	8.0 ± 0.2	639 ± 86	0.4 ± 0.2	299 ± 16
	Hot	2.2 × 10^2^ ± 2.9 × 10^1^	1.1 × 10^2^ ± 1.4 × 10^1^	BD	51.9 ± 16.3	7.8 ± 0.2	700 ± 30	NA	NA
C	Cold	1.5 × 10^4^ ± 2.0 × 10^2^	1.3 × 10^3^ ± 2.0 × 10^2^	BD	23.1 ± 2.5	8.0 ± 0	707 ± 60	0.5 ± 0	299 ± 16
	Hot	NA	NA	NA	NA	NA	NA	NA	NA
D	Cold	1.6 × 10^5^ ± 8.7 × 10^3^	1.2 × 10^4^ ± 8.9 × 10^3^	BD	22.5 ± 1.6	7.6 ± 0.1	509 ± 118	0.7 ± 0.35	229 ± 26
	Hot	NA	NA	NA	NA	NA	NA	NA	NA
E	Cold	1.7 × 10^4^ ± 2.0 × 10^2^	1.5 × 10^3^ ± 2.0 × 10^1^	BD	25.2 ± 5.2	7.8 ± 0.3	699 ± 82	0.2 ± 0.05	269 ± 11
	Hot	1.6 × 10^4^ ± 3.5 × 10^2^	4.1 × 10^2^ ± 4.2 × 10^1^	BD	47.9 ± 5.1	7.8 ± 0.2	761 ± 30	NA	NA
F	Cold	1.1 × 10^5^ ± 1.5 × 10^2^	6.3 × 10^4^ ± 7.1 × 10^2^	1.5 × 10^2^ ± 2.3 × 10^2^	24.7 ± 2.9	7.8 ± 0.2	556 ± 106	0.4 ± 0.2	261 ± 3
	Hot	2.5 × 10^4^ ± 2.6 × 10^2^	3.2 × 10^3^ ± 3.3 × 10^1^	9.1 × 10^1^ ± 1.3 × 10^2^	43.3 ± 5.7	7.8 ± 0.2	583 ± 93	NA	NA
G	Cold	2.9 × 10^4^ ± 2.0 × 10^2^	2.2 × 10^3^ ± 2.2 × 10^2^	8.3 × 10^0^ ± 2.0 × 10^1^	21.6 ± 2.7	8.0 ± 0.3	648 ± 179	0.4 ± 0.2	266 ± 19
	Hot	1.8 × 10^4^ ± 1.5 × 10^2^	2.7 × 10^3^ ± 1.8 × 10^1^	BD	38.6 ± 6.8	8.0 ± 0.3	596 ± 190	NA	NA
H	Cold	2.7 × 10^4^ ± 2.5 × 10^2^	1.4 × 10^3^ ± 1.3 × 10^2^	BD	20.8 ± 1.3	7.8 ± 0.1	707 ± 191	0.4 ± 0.2	266 ± 19
	Hot	NA	NA	NA	NA	NA	NA	NA	NA

NA: Not Available. BD: Below detection limit (<5 CFU/L).

**Table 2:** Occurrence frequencies of *Legionella* in water and biofilm samples obtained from eight hospitals and Al-Quds University in the West Bank during the study period (2012–2014).

	CDA ^1^			CIA ^2^			
Year	*L. pneumo-phila* Isolates/Total Number ^3^	% of Isolates	*Leg*. Counts (Mean of Cold Water) (CFU/l)±SD	*Legionella* spp (Lgsp) Positive Samples/Total Number	% of Positive Samples	*L. pneumo-phila* (Lpn) Positive Samples/Total Number	% of Positive Samples
2012	96/409	23.5	NA	43/53	81.1	36/53	67.9
2013	30/346	8.7	NA	64/106	60.4	55/106	51.9
2014	71/453	15.7	NA	102/138	73.9	79/138	57.2
**Hospital**							
A	30/150	20	4.3 × 10^1^ ± 1.1 × 10^2^	29/36	80.6	23/36	63.9
B	35/156	22.4	BD	28/42	66.7	20/42	47.6
C	5/150	3.3	BD	19/36	52.8	11/36	30.6
D	18/90	20	BD	16/21	76.2	13/21	61.9
E	11/156	7.1	BD	17/42	40.5	13/42	31
F	41/156	26.3	1.5 × 10^2^ ± 2.3 × 10^2^	37/42	88.1	34/42	81
G	35/156	22.4	8.3 × 10^0^ ± 2.0 × 10^2^	39/42	92.9	38/42	90.5
H	6/150	4	BD	24/36	66.7	18/36	50
AQU	16/44	36.4	NA	NA	NA	NA	NA
**Sample type**							
Water	6/72	8.3		42/72	58.3	36/72	50
Biofilm	191/1136	16.8		167/225	74.2	134/225	59.5

NA: Not Available; BD: Below detection limit (<5 CFU/L); AQU: Al-Quds University; **^1^** CDA: Cultivation Dependent Analysis; **^2^** CIA: Cultivation Independent Analysis; **^3^** The number of isolates corresponds to the number *L. pneumophila* culture-positive water and biofilm samples.

**Table 3:** VNTR characteristics of the *L. pneumophila* strains isolated from the West Bank.

	West Bank		
VNTR	No. of Repeats	HGDI ^1^ (CI 95%)	Null Alleles (%)
Lpms1	4	0.528 (0.459–0.596)	0
Lpms3	2	0.461 (0.420–0.502)	0
Lpms13	5	0.579 (0.506–0.652)	0
Lpms17	2	0.115 (0.053–0.178)	0
Lpms19	2	0.022 (1.000–0.053)	1.11
Lpms31	6	0.576 (0.513–0.639)	1.67
Lpms33	4	0.575 (0.506–0.643)	0
Lpms34	4	0.503 (0.429–0.577)	2.22
Lpms35	6	0.687 (0.641–0.733)	1.67
Lpms38	3	0.249 (0.168–0.330)	4.44
Lpms39	3	0.509 (0.445–0.574)	0
Lpms40	3	0.493 (0.444–0.541)	3.33
Lpms44	3	0.498 (0.463–0.533)	0

**^1^** HGDI: Hunter-Gaston Discrimination Index.

**Table 4:** Serogroup and monoclonal antibody subtyping of 180 environmental *L. pneumophila* isolates from the West Bank.

Serogroup	mAb ^1^ Subgroup	*L. pneumophila* Isolates	
		No.	Frequency (%)
1	OLDA	10	5.6
1	NA ^2^	101	56.1
*Total Sg1*		*111*	*61.6*
6	Dresden	54	30.0
8	NA ^2^	6	3.3
10	NA ^2^	2	1.1
(2–14)	NA ^2^	7	3.9
*Total non-Sg1*		*69*	*38.3*
Total		180	100

^1^ mAb: monoclonal Antibody; ^2^ NA: Not analyzed.

**Table 5:** MLVA-8(12) genotype abundances at the sampling sites in the West Bank.

Location	MLVA-8 (12)-Genotype	No of Strains per Genotype (%)	Sg-MAb	MLVA- Clonal Complex (VACC)	No. of Strains per VACC (%)
Hospital A	Gt4(17)	20 (71)	1	VACC1	20(71)
	Gt16(1)	4 (14)	6 Dresden	VACC5	5(18)
	Gt13(72)	3 (11)	6 Dresden	VACC2	3(11)
	Gt16(6)	1 (4)	(2-14)	0	0
	*Total*	*28(100)*	*0*	*0*	*28(100)*
Hospital B	Gt4(17)	21(66)	1	VACC1	24(75)
	Gt9(92)	7(22)	6 Dresden	VACC11	7(22)
	Gt63(83)	2(6)	1	VACC2	1(3)
	Gt4(16)	1(3)	1	0	0
	Gt13(72)	1(3)	6 Dresden	0	0
	*Total*	*32(100)*	*0*	*0*	*32(100)*
Hospital C	Gt4(17)	4(80)	1	VACC1	4(80)
	Gt10(93)	1(20)	6 Dresden	VACC11	1(20)
	*Total*	*5(100)*	*0*	*0*	*5(100)*
Hospital D	Gt4(17)	15(83)	1	VACC1	15(83)
	Gt13(72)	3(17)	6 Dresden	VACC2	3(17)
	*Total*	*18(100)*	*0*	*0*	*18(100)*
Hospital E	Gt13(143)	2(20)	10	VACC2	5(50)
	Gt64(72)	2(20)	6 Dresden	VACC5	3(30)
	Gt8(7)	2(20)	(2-14)	VACC1	2(20)
	Gt4(17)	2(20)	1	0	0
	Gt24(68)	1(10)	(2-14)	0	0
	Gt16(3)	1(10)	(2-14)	0	0
	*Total*	*10(100)*	*0*	*0*	*10(100)*
Hospital F	Gt10(93)	14(44)	6 Dresden	VACC11	21(66)
	Gt10(141)	6(19)	6 Dresden	VACC1	6(19)
	Gt4(17)	6(19)	1	VACC2	5(16)
	Gt64(74)	3(9)	6 Dresden	0	0
	Gt55(94)	1(3)	6 Dresden	0	0
	Gt64(72)	1(3)	6 Dresden	0	0
	Gt38(109)	1(3)	1	0	0
	*Total*	*32(100)*	*0*	*0*	*32(100)*
Hospital G	Gt6(18)	30(88)	1	VACC1	31(91)
	Gt6(15)	1(3)	1	VACC5	3(9)
	Gt16(1)	1(3)	6 Dresden	0	0
	Gt8(142)	1(3)	(2-14)	0	0
	Gt8(23)	1(3)	(2-14)	0	0
	*Total*	*34(100)*	*0*	*0*	*34(100)*
Hospital H	Gt40(47)	3(50)	6 Dresden	VACC5	3(50)
	Gt10(93)	1(17)	6 Dresden	VACC11	2(33)
	Gt9(92)	1(17)	6	VACC1	1(17)
	Gt63(83)	1(17)	1	0	0
	*Total*	*6(100)*	*0*	*0*	*6(100)*
AQU *	Gt4(17)	6(40)	1 OLDA	VACC1	13(87)
	Gt12(84)	5(33)	8	VACC2	2(13)
	Gt4(20)	1(7)	1 OLDA	0	0
	Gt11(87)	1(7)	8	0	0
	Gt13(106)	1(7)	6	0	0
	Gt84(106)	1(7)	6	0	0
	*Total*	*15(100)*	*0*	*0*	*15(100)*

*: Al-Quds University.

## Supplementary Materials

The following are available online at https://www.mdpi.com/2076-0817/9/11/862/s1, Table S1. List of *L. pneumophila* strains (n = 180) isolated from the West Bank analyzed in this study, MLVA-8(12) Figure S1: Sampling map of the eight hospitals and Al-Quds University in the West Bank. Figure S2: UPGMA based clustering analysis of the MLVA-8(12) profiles of 180 *L. pneumophila* strains isolated from water and biofilm samples of the Al-Quds University campus and eight hospitals of the West Bank.
