# Supplementary material for: Characterization of Legionella pneumophila Populations by Multilocus Variable Number of Tandem Repeats (MLVA) Genotyping from Drinking Water and Biofilm in Hospitals from Different Regions of the West Bank
Source: Pathogens. 2020 Oct 22;9(11):862. doi: 10.3390/pathogens9110862 (PMC7690423; doi:10.3390/pathogens9110862)
Supplement: Supplementary file 1 [file pathogens-09-00862-s001.pdf]

Supplementary Material:

Zayed et al., *Legionella pneumophila* MLVA-genotypes of the West Bank

Table S1. List of *L. pneumophila* strains (n=180) isolated from the West Bank analysed in this study by MLVA-8(12). MLVA profiles of isolates from water are highlighted in bold.

Strain desig.	Sg, Mab[1]	ST	Lpn PCR	MLVA-8(12) - genotype	MLVA-8(12) profile[3]	MLVA-Clonal Complex	Location	Sample type	Year
A1	1 OLDA	1	+	Gt4(17)	7,7,10,2,4,9,4,2,17,3,14,5,5	VACC1	Hospital D	Biofilm	2012
A2	1 OLDA	1	+	Gt4(17)	7,7,10,2,4,9,4,2,17,3,14,5,5	VACC1	Hospital D	Biofilm	2012
A3	1 OLDA	1	+	Gt4(20)	7,7,10,2,4,9,4,2,17,3,14,4,5	VACC1	AQU	Biofilm	2012
A4	1 OLDA	1	+	Gt4(17)	7,7,10,2,4,9,4,2,17,3,14,5,5	VACC1	AQU	Biofilm	2012
A5	1 OLDA	1	+	Gt4(17)	7,7,10,2,4,9,4,2,17,3,14,5,5	VACC1	AQU	Biofilm	2012
A6	1 OLDA	1	+	Gt4(17)	7,7,10,2,4,9,4,2,17,3,14,5,5	VACC1	Hospital D	Biofilm	2012
A7	8	1358	+	Gt11(87)	7,7,10,2,4,17,1,1,18,3,14,5,8	VACC1	AQU	Biofilm	2012
A8	6	187	+	Gt84(106)	8,8,11,1,0,16,1,1,3,3,6,4,8	VACC2	AQU	Biofilm	2012
A9	1 OLDA	1	+	Gt4(17)	7,7,10,2,4,9,4,2,17,3,14,5,5	VACC1	AQU	Biofilm	2012
A10	6	1326	+	Gt13(106)	8,8,11,1,4,16,1,1,3,3,6,4,8	VACC2	AQU	Biofilm	2012
A12	1 OLDA	1	+	Gt4(17)	7,7,10,2,4,9,4,2,17,3,14,5,5	VACC1	AQU	Biofilm	2012
A13	1 OLDA	1	+	Gt4(17)	7,7,10,2,4,9,4,2,17,3,14,5,5	VACC1	AQU	Biofilm	2012
A14	8	1358	+	Gt12(84)	7,7,9,2,4,17,1,1,18,0,14,5,8	VACC1	AQU	Biofilm	2012
A15	8	1358	+	Gt12(84)	7,7,9,2,4,17,1,1,18,0,14,5,8	VACC1	AQU	Biofilm	2012
A16	8	1358	+	Gt12(84)	7,7,9,2,4,17,1,1,18,0,14,5,8	VACC1	AQU	Biofilm	2012
A17	8	1358	+	Gt12(84)	7,7,9,2,4,17,1,1,18,0,14,5,8	VACC1	AQU	Biofilm	2012
A18	8	1358	+	Gt12(84)	7,7,9,2,4,17,1,1,18,0,14,5,8	VACC1	AQU	Biofilm	2012
A19	6	461	+	Gt10(93)	9,8,6,2,4,13,3,4,10,3,10,4,8	VACC11	Hospital F	Biofilm	2012
A20	1	1	+	Gt4(17)	7,7,10,2,4,9,4,2,17,3,14,5,5	VACC1	Hospital F	Biofilm	2012

**A21**	**6**	**461**	**+**	**Gt10(93)**	**9,8,6,2,4,13,3,4,10,3,10,4,8**	**VACC11**	**Hospital F**	**Water**	**2012**
A22	6	461	+	Gt9(92)	9,8,11,2,4,13,3,4,17,3,10,4,8	VACC11	Hospital H	Biofilm	2012
A23	6	1438	+	Gt16(1)	9,8,8,2,4,13,2,2,18,3,10,4,8	VACC5	Hospital G	Biofilm	2012
A24	(2-14)	1482	+	Gt8(23)	10,8,8,2,4,13,2,2,18,3,14,4,8	VACC5	Hospital G	Biofilm	2012
A25	1	1	+	Gt6(18)	7,7,10,2,4,9,4,2,18,3,14,5,5	VACC1	Hospital G	Biofilm	2012
A26	1	1	+	Gt6(18)	7,7,10,2,4,9,4,2,18,3,14,5,5	VACC1	Hospital G	Biofilm	2012
A27	1	1	+	Gt6(18)	7,7,10,2,4,9,4,2,18,3,14,5,5	VACC1	Hospital G	Biofilm	2012
A28	1	1	+	Gt6(18)	7,7,10,2,4,9,4,2,18,3,14,5,5	VACC1	Hospital G	Biofilm	2012
A29	1	1	+	Gt6(18)	7,7,10,2,4,9,4,2,18,3,14,5,5	VACC1	Hospital G	Biofilm	2012
A30	1	1	+	Gt6(18)	7,7,10,2,4,9,4,2,18,3,14,5,5	VACC1	Hospital G	Biofilm	2012
A31	1	1	+	Gt6(18)	7,7,10,2,4,9,4,2,18,3,14,5,5	VACC1	Hospital G	Biofilm	2012
**A32**	**1**	**1**	**+**	**Gt6(18)**	**7,7,10,2,4,9,4,2,18,3,14,5,5**	**VACC1**	**Hospital G**	**Water**	**2012**
A33	1	1	+	Gt6(18)	7,7,10,2,4,9,4,2,18,3,14,5,5	VACC1	Hospital G	Biofilm	2012
A34	6	1438	+	Gt16(1)	9,8,8,2,4,13,2,2,18,3,10,4,8	VACC5	Hospital A	Biofilm	2012
A35	1	1	+	Gt4(17)	7,7,10,2,4,9,4,2,17,3,14,5,5	VACC1	Hospital A	Biofilm	2012
A36	(2-14)	1438	+	Gt16(6)	9,8,8,2,4,13,2,2,18,2,10,4,8	VACC5	Hospital A	Biofilm	2012
A37	6	1326	+	Gt13(72)	8,8,11,1,4,16,1,1,3,2,6,4,8	VACC2	Hospital A	Biofilm	2012
A38	6	1438	+	Gt16(1)	9,8,8,2,4,13,2,2,18,3,10,4,8	VACC5	Hospital A	Biofilm	2012
A39	1	1	+	Gt4(17)	7,7,10,2,4,9,4,2,17,3,14,5,5	VACC1	Hospital A	Biofilm	2012
A40	1	1	+	Gt4(17)	7,7,10,2,4,9,4,2,17,3,14,5,5	VACC1	Hospital A	Biofilm	2012
A41	6	1326	+	Gt13(72)	8,8,11,1,4,16,1,1,3,2,6,4,8	VACC2	Hospital A	Biofilm	2012
A42	1	1	+	Gt4(17)	7,7,10,2,4,9,4,2,17,3,14,5,5	VACC1	Hospital A	Biofilm	2012
A43	1	1	+	Gt4(17)	7,7,10,2,4,9,4,2,17,3,14,5,5	VACC1	Hospital A	Biofilm	2012
A44	6	1326	+	Gt13(72)	8,8,11,1,4,16,1,1,3,2,6,4,8	VACC2	Hospital A	Biofilm	2012
A45	1	1	+	Gt4(17)	7,7,10,2,4,9,4,2,17,3,14,5,5	VACC1	Hospital A	Biofilm	2012

A46	6 Dresden	1438	+	Gt16(1)	9,8,8,2,4,13,2,2,18,3,10,4,8	VACC5	Hospital A	Biofilm	2012
A47	1	1	+	Gt4(17)	7,7,10,2,4,9,4,2,17,3,14,5,5	VACC1	Hospital A	Biofilm	2012
A48	1	1	+	Gt4(17)	7,7,10,2,4,9,4,2,17,3,14,5,5	VACC1	Hospital A	Biofilm	2012
A49	1	1	+	Gt4(17)	7,7,10,2,4,9,4,2,17,3,14,5,5	VACC1	Hospital A	Biofilm	2012
**A50**	**6**	**1438**	**+**	**Gt16(1)**	**9,8,8,2,4,13,2,2,18,3,10,4,8**	**VACC5**	**Hospital A**	**Water**	**2012**
A51	1	1	+	Gt4(17)	7,7,10,2,4,9,4,2,17,3,14,5,5	VACC1	Hospital A	Biofilm	2012
A52	1	1	+	Gt4(17)	7,7,10,2,4,9,4,2,17,3,14,5,5	VACC1	Hospital A	Biofilm	2012
A53	1	1	+	Gt4(17)	7,7,10,2,4,9,4,2,17,3,14,5,5	VACC1	Hospital A	Biofilm	2012
A54	1	1	+	Gt4(17)	7,7,10,2,4,9,4,2,17,3,14,5,5	VACC1	Hospital A	Biofilm	2012
A55	1	1	+	Gt4(17)	7,7,10,2,4,9,4,2,17,3,14,5,5	VACC1	Hospital A	Biofilm	2012
A56	1	1	+	Gt4(17)	7,7,10,2,4,9,4,2,17,3,14,5,5	VACC1	Hospital D	Biofilm	2012
A57	1	1	+	Gt4(17)	7,7,10,2,4,9,4,2,17,3,14,5,5	VACC1	Hospital D	Biofilm	2012
A58	1	1	+	Gt4(17)	7,7,10,2,4,9,4,2,17,3,14,5,5	VACC1	Hospital D	Biofilm	2012
A59	1	1	+	Gt4(17)	7,7,10,2,4,9,4,2,17,3,14,5,5	VACC1	Hospital D	Biofilm	2012
A60	1	1	+	Gt4(17)	7,7,10,2,4,9,4,2,17,3,14,5,5	VACC1	Hospital D	Biofilm	2012
A61	1	1	+	Gt4(17)	7,7,10,2,4,9,4,2,17,3,14,5,5	VACC1	Hospital D	Biofilm	2012
A62	1	1	+	Gt4(17)	7,7,10,2,4,9,4,2,17,3,14,5,5	VACC1	Hospital D	Biofilm	2012
A63	1	1	+	Gt4(17)	7,7,10,2,4,9,4,2,17,3,14,5,5	VACC1	Hospital D	Biofilm	2012
A64	1	1	+	Gt4(17)	7,7,10,2,4,9,4,2,17,3,14,5,5	VACC1	Hospital D	Biofilm	2012
A65	1	1	+	Gt4(17)	7,7,10,2,4,9,4,2,17,3,14,5,5	VACC1	Hospital D	Biofilm	2012
A66	1	1	+	Gt4(17)	7,7,10,2,4,9,4,2,17,3,14,5,5	VACC1	Hospital D	Biofilm	2012
A67	1	1	+	Gt4(17)	7,7,10,2,4,9,4,2,17,3,14,5,5	VACC1	Hospital D	Biofilm	2012
A68	6	1326	+	Gt13(72)	8,8,11,1,4,16,1,1,3,2,6,4,8	VACC2	Hospital D	Biofilm	2012
A69	6	1326	+	Gt13(72)	8,8,11,1,4,16,1,1,3,2,6,4,8	VACC2	Hospital D	Biofilm	2012
A70	6	1326	+	Gt13(72)	8,8,11,1,4,16,1,1,3,2,6,4,8	VACC2	Hospital D	Biofilm	2012

A71	6	461	+	Gt10(93)	9,8,6,2,4,13,3,4,10,3,10,4,8	VACC11	Hospital C	Biofilm	2012
A72	1	1	+	Gt4(17)	7,7,10,2,4,9,4,2,17,3,14,5,5	VACC1	Hospital C	Biofilm	2012
A73	1	1	+	Gt4(17)	7,7,10,2,4,9,4,2,17,3,14,5,5	VACC1	Hospital C	Biofilm	2012
A74	1	1	+	Gt4(17)	7,7,10,2,4,9,4,2,17,3,14,5,5	VACC1	Hospital B	Biofilm	2012
A75	1	1	+	Gt4(17)	7,7,10,2,4,9,4,2,17,3,14,5,5	VACC1	Hospital B	Biofilm	2012
A76	1	1	+	Gt4(17)	7,7,10,2,4,9,4,2,17,3,14,5,5	VACC1	Hospital B	Biofilm	2012
A77	1	1	+	Gt4(16)	7,7,10,2,4,9,4,2,17,2,14,5,5	VACC1	Hospital B	Biofilm	2012
A78	1	1	+	Gt4(17)	7,7,10,2,4,9,4,2,17,3,14,5,5	VACC1	Hospital B	Biofilm	2012
A79	6	1326	+	Gt13(72)	8,8,11,1,4,16,1,1,3,2,6,4,8	VACC2	Hospital B	Biofilm	2012
A80	1	1	+	Gt4(17)	7,7,10,2,4,9,4,2,17,3,14,5,5	VACC1	Hospital B	Biofilm	2012
A81	1	1	+	Gt4(17)	7,7,10,2,4,9,4,2,17,3,14,5,5	VACC1	Hospital B	Biofilm	2012
A82	6	461	+	Gt9(92)	9,8,11,2,4,13,3,4,17,3,10,4,8	VACC11	Hospital B	Biofilm	2012
A83	1	1	+	Gt4(17)	7,7,10,2,4,9,4,2,17,3,14,5,5	VACC1	Hospital B	Biofilm	2012
A84	6	461	+	Gt9(92)	9,8,11,2,4,13,3,4,17,3,10,4,8	VACC11	Hospital B	Biofilm	2012
A85	1	1	+	Gt4(17)	7,7,10,2,4,9,4,2,17,3,14,5,5	VACC1	Hospital B	Biofilm	2012
A86	6	461	+	Gt9(92)	9,8,11,2,4,13,3,4,17,3,10,4,8	VACC11	Hospital B	Biofilm	2012
A87	1	1	+	Gt4(17)	7,7,10,2,4,9,4,2,17,3,14,5,5	VACC1	Hospital B	Biofilm	2012
A88	1	1	+	Gt4(17)	7,7,10,2,4,9,4,2,17,3,14,5,5	VACC1	Hospital B	Biofilm	2012
A89	1	1	+	Gt4(17)	7,7,10,2,4,9,4,2,17,3,14,5,5	VACC1	Hospital B	Biofilm	2012
A90	1	1	+	Gt4(17)	7,7,10,2,4,9,4,2,17,3,14,5,5	VACC1	Hospital B	Biofilm	2012
A91	(2-14)	1482	+	Gt8(7)	10,8,8,2,4,13,2,2,18,2,10,4,8	VACC5	Hospital E	Biofilm	2012
A92	(2-14)	1482	+	Gt8(7)	10,8,8,2,4,13,2,2,18,2,10,4,8	VACC5	Hospital E	Biofilm	2012
A93	(2-14)	93	+	Gt24(68)	8,8,11,2,0,16,1,1,3,0,6,4,8	VACC2	Hospital E	Biofilm	2012
A94	(2-14)	1438	+	Gt16(3)	9,8,8,2,4,13,2,2,18,2,10,5,8	VACC5	Hospital E	Biofilm	2012
A95	6 Dresden	9	+	Gt64(72)	8,8,11,2,4,16,1,1,3,2,6,4,8	VACC2	Hospital E	Biofilm	2012

A97	6	461	+	Gt10(93)	9,8,6,2,4,13,3,4,10,3,10,4,8	VACC11	Hospital F	Biofilm	2012
A98	6 Dresden	9	+	Gt64(72)	8,8,11,2,4,16,1,1,3,2,6,4,8	VACC2	Hospital F	Biofilm	2012
A99	6 Dresden	461	+	Gt10(93)	9,8,6,2,4,13,3,4,10,3,10,4,8	VACC11	Hospital F	Biofilm	2012
A100	1	NA	+	Gt38(109)	8,8,6,2,4,8,1,1,3,2,6,4,8	VACC2	Hospital F	Biofilm	2012
A101	1	NA	+	Gt63(83)	7,7,10,2,4,0,4,0,0,3,14,4,9	VACC1	Hospital H	Biofilm	2012
A102	1	NA	+	Gt63(83)	7,7,10,2,4,0,4,0,0,3,14,4,9	VACC1	Hospital B	Biofilm	2012
A103	1	NA	+	Gt63(83)	7,7,10,2,4,0,4,0,0,3,14,4,9	VACC1	Hospital H	Biofilm	2012
A104	1	1	+	Gt4(17)	7,7,10,2,4,9,4,2,17,3,14,5,5	VACC1	Hospital B	Biofilm	2013
A105	1	1	+	Gt4(17)	7,7,10,2,4,9,4,2,17,3,14,5,5	VACC1	Hospital B	Biofilm	2013
A106	1	1	+	Gt6(15)	7,7,10,2,4,9,4,2,18,2,14,5,5	VACC1	Hospital G	Biofilm	2013
A107	6	461	+	Gt9(92)	9,8,11,2,4,13,3,4,17,3,10,4,8	VACC11	Hospital B	Biofilm	2013
A108	6 Dresden	461	+	Gt10(93)	9,8,6,2,4,13,3,4,10,3,10,4,8	VACC11	Hospital F	Biofilm	2013
A109	1	1	+	Gt4(17)	7,7,10,2,4,9,4,2,17,3,14,5,5	VACC1	Hospital F	Biofilm	2013
A110	6 Dresden	461	+	Gt10(141)	9,8,6,2,4,13,3,4,10,3,10,0,8	VACC11	Hospital F	Biofilm	2013
A112	6 Dresden	461	+	Gt10(93)	9,8,6,2,4,13,3,4,10,3,10,4,8	VACC11	Hospital F	Biofilm	2013
**A114**	**6 Dresden**	**461**	**+**	**Gt10(93)**	**9,8,6,2,4,13,3,4,10,3,10,4,8**	**VACC11**	**Hospital F**	**Water**	**2013**
A115	(2-14)	461	+	Gt10(93)	9,8,6,2,4,13,3,4,10,3,10,4,8	VACC11	Hospital F	Biofilm	2013
A116	6 Dresden	461	+	Gt10(141)	9,8,6,2,4,13,3,4,10,3,10,0,8	VACC11	Hospital F	Biofilm	2013
A119	6 Dresden	9	+	Gt64(74)	8,8,11,2,4,16,1,1,3,3,6,4,8	VACC2	Hospital F	Biofilm	2013
A121	6 Dresden	NA	+	Gt55(94)	9,8,6,2,4,13,3,0,10,3,10,4,8	VACC11	Hospital F	Biofilm	2013
A122	1	1	+	Gt4(17)	7,7,10,2,4,9,4,2,17,3,14,5,5	VACC1	Hospital D	Biofilm	2013
A123	1	1	+	Gt4(17)	7,7,10,2,4,9,4,2,17,3,14,5,5	VACC1	Hospital B	Biofilm	2013

A124	1	1	+	Gt4(17)	7,7,10,2,4,9,4,2,17,3,14,5,5	VACC1	Hospital B	Biofilm	2013
A127	6 Dresden	461	+	Gt10(93)	9,8,6,2,4,13,3,4,10,3,10,4,8	VACC11	Hospital F	Biofilm	2013
A128	6 Dresden	461	+	Gt10(93)	9,8,6,2,4,13,3,4,10,3,10,4,8	VACC11	Hospital F	Biofilm	2013
A129	6 Dresden	9	+	Gt64(74)	8,8,11,2,4,16,1,1,3,3,6,4,8	VACC2	Hospital F	Biofilm	2013
A130	1	1	+	Gt6(18)	7,7,10,2,4,9,4,2,18,3,14,5,5	VACC1	Hospital G	Biofilm	2013
A131	1	1	+	Gt6(18)	7,7,10,2,4,9,4,2,18,3,14,5,5	VACC1	Hospital G	Biofilm	2013
A132	1	1	+	Gt6(18)	7,7,10,2,4,9,4,2,18,3,14,5,5	VACC1	Hospital G	Biofilm	2013
A133	1	1	+	Gt6(18)	7,7,10,2,4,9,4,2,18,3,14,5,5	VACC1	Hospital G	Biofilm	2013
A134	1	1	+	Gt6(18)	7,7,10,2,4,9,4,2,18,3,14,5,5	VACC1	Hospital G	Biofilm	2013
A135	1	1	+	Gt6(18)	7,7,10,2,4,9,4,2,18,3,14,5,5	VACC1	Hospital G	Biofilm	2013
A137	1	1	+	Gt4(17)	7,7,10,2,4,9,4,2,17,3,14,5,5	VACC1	Hospital B	Biofilm	2013
A138	6 Dresden	461	+	Gt9(92)	9,8,11,2,4,13,3,4,17,3,10,4,8	VACC11	Hospital B	Biofilm	2013
A139	1	1	+	Gt4(17)	7,7,10,2,4,9,4,2,17,3,14,5,5	VACC1	Hospital B	Biofilm	2013
A140	1	1	+	Gt4(17)	7,7,10,2,4,9,4,2,17,3,14,5,5	VACC1	Hospital B	Biofilm	2013
A141	1	1	+	Gt4(17)	7,7,10,2,4,9,4,2,17,3,14,5,5	VACC1	Hospital B	Biofilm	2013
A142	1	1	+	Gt4(17)	7,7,10,2,4,9,4,2,17,3,14,5,5	VACC1	Hospital E	Biofilm	2013
A143	1	1	+	Gt4(17)	7,7,10,2,4,9,4,2,17,3,14,5,5	VACC1	Hospital E	Biofilm	2013
A144	6	9	+	Gt64(72)	8,8,11,2,4,16,1,1,3,2,6,4,8	VACC2	Hospital E	Biofilm	2013
A145	1	1	+	Gt4(17)	7,7,10,2,4,9,4,2,17,3,14,5,5	VACC1	Hospital B	Biofilm	2013
**A148**	**6 Dresden**	**461**	**+**	**Gt10(141)**	**9,8,6,2,4,13,3,4,10,3,10,0,8**	**VACC11**	**Hospital F**	**Water**	**2013**
A149	6 Dresden	461	+	Gt10(141)	9,8,6,2,4,13,3,4,10,3,10,0,8	VACC11	Hospital F	Biofilm	2013
A152	6 Dresden	461	+	Gt10(93)	9,8,6,2,4,13,3,4,10,3,10,4,8	VACC11	Hospital F	Biofilm	2013
A153	6 Dresden	461	+	Gt10(93)	9,8,6,2,4,13,3,4,10,3,10,4,8	VACC11	Hospital F	Biofilm	2013

A154	1 OLDA	1	+	Gt4(17)	7,7,10,2,4,9,4,2,17,3,14,5,5	VACC1	Hospital F	Biofilm	2013
A156	6 Dresden	9	+	Gt64(74)	8,8,11,2,4,16,1,1,3,3,6,4,8	VACC2	Hospital F	Biofilm	2013
A157	6 Dresden	461	+	Gt10(141)	9,8,6,2,4,13,3,4,10,3,10,0,8	VACC11	Hospital F	Biofilm	2013
A159	6 Dresden	461	+	Gt10(141)	9,8,6,2,4,13,3,4,10,3,10,0,8	VACC11	Hospital F	Biofilm	2013
A161	1	1	+	Gt4(17)	7,7,10,2,4,9,4,2,17,3,14,5,5	VACC1	Hospital F	Biofilm	2013
A162	1	1	+	Gt6(18)	7,7,10,2,4,9,4,2,18,3,14,5,5	VACC1	Hospital G	Biofilm	2013
A163	1	1	+	Gt6(18)	7,7,10,2,4,9,4,2,18,3,14,5,5	VACC1	Hospital G	Biofilm	2013
A164	1	1	+	Gt6(18)	7,7,10,2,4,9,4,2,18,3,14,5,5	VACC1	Hospital G	Biofilm	2013
A165	1	1	+	Gt6(18)	7,7,10,2,4,9,4,2,18,3,14,5,5	VACC1	Hospital G	Biofilm	2013
A166	(2-14)	1482	+	Gt8(142)	10,8,8,2,4,13,2,2,18,3,10,4,8	VACC5	Hospital G	Biofilm	2013
A167	1	1	+	Gt6(18)	7,7,10,2,4,9,4,2,18,3,14,5,5	VACC1	Hospital G	Biofilm	2013
A168	1	1	+	Gt6(18)	7,7,10,2,4,9,4,2,18,3,14,5,5	VACC1	Hospital G	Biofilm	2013
A169	1	1	+	Gt4(17)	7,7,10,2,4,9,4,2,17,3,14,5,5	VACC1	Hospital A	Biofilm	2014
A170	1	1	+	Gt4(17)	7,7,10,2,4,9,4,2,17,3,14,5,5	VACC1	Hospital A	Biofilm	2014
A171	1	1	+	Gt4(17)	7,7,10,2,4,9,4,2,17,3,14,5,5	VACC1	Hospital A	Biofilm	2014
A172	1	1	+	Gt4(17)	7,7,10,2,4,9,4,2,17,3,14,5,5	VACC1	Hospital A	Biofilm	2014
A173	1	1	+	Gt4(17)	7,7,10,2,4,9,4,2,17,3,14,5,5	VACC1	Hospital A	Biofilm	2014
A174	1	1	+	Gt4(17)	7,7,10,2,4,9,4,2,17,3,14,5,5	VACC1	Hospital A	Biofilm	2014
A175	1	1	+	Gt4(17)	7,7,10,2,4,9,4,2,17,3,14,5,5	VACC1	Hospital C	Biofilm	2014
A176	1	1	+	Gt4(17)	7,7,10,2,4,9,4,2,17,3,14,5,5	VACC1	Hospital C	Biofilm	2014
A177	6	461	+	Gt9(92)	9,8,11,2,4,13,3,4,17,3,10,4,8	VACC11	Hospital B	Biofilm	2014
A178	6 Dresden	461	+	Gt9(92)	9,8,11,2,4,13,3,4,17,3,10,4,8	VACC11	Hospital B	Biofilm	2014
A179	1	1	+	Gt4(17)	7,7,10,2,4,9,4,2,17,3,14,5,5	VACC1	Hospital B	Biofilm	2014
A180	1	1	+	Gt6(18)	7,7,10,2,4,9,4,2,18,3,14,5,5	VACC1	Hospital G	Biofilm	2014

A181	1	1	+	Gt4(17)	7,7,10,2,4,9,4,2,17,3,14,5,5	VACC1	Hospital F	Biofilm	2014
A182	6 Dresden	461	+	Gt10(93)	9,8,6,2,4,13,3,4,10,3,10,4,8	VACC11	Hospital F	Biofilm	2014
A183	6 Dresden	461	+	Gt10(93)	9,8,6,2,4,13,3,4,10,3,10,4,8	VACC11	Hospital F	Biofilm	2014
A184	1	1	+	Gt6(18)	7,7,10,2,4,9,4,2,18,3,14,5,5	VACC1	Hospital G	Biofilm	2014
A186	1	1	+	Gt6(18)	7,7,10,2,4,9,4,2,18,3,14,5,5	VACC1	Hospital G	Biofilm	2014
A187	1	1	+	Gt6(18)	7,7,10,2,4,9,4,2,18,3,14,5,5	VACC1	Hospital G	Biofilm	2014
A188	1	1	+	Gt6(18)	7,7,10,2,4,9,4,2,18,3,14,5,5	VACC1	Hospital G	Biofilm	2014
A189	1	1	+	Gt6(18)	7,7,10,2,4,9,4,2,18,3,14,5,5	VACC1	Hospital G	Biofilm	2014
A190	1	1	+	Gt6(18)	7,7,10,2,4,9,4,2,18,3,14,5,5	VACC1	Hospital G	Biofilm	2014
A191	1	1	+	Gt6(18)	7,7,10,2,4,9,4,2,18,3,14,5,5	VACC1	Hospital G	Biofilm	2014
A192	1	1	+	Gt6(18)	7,7,10,2,4,9,4,2,18,3,14,5,5	VACC1	Hospital G	Biofilm	2014
A193	6 Dresden	292	+	Gt40(47)	8,8,9,2,4,13,2,2,21,3,10,4,8	VACC5	Hospital H	Biofilm	2014
A194	6 Dresden	292	+	Gt40(47)	8,8,9,2,4,13,2,2,21,3,10,4,8	VACC5	Hospital H	Biofilm	2014
A195	6 Dresden	292	+	Gt40(47)	8,8,9,2,4,13,2,2,21,3,10,4,8	VACC5	Hospital H	Biofilm	2014
A196	10	1326	+	Gt13(143)	8,8,11,1,4,16,1,1,3,0,6,4,8	VACC2	Hospital E	Biofilm	2014
A197	10	1326	+	Gt13(143)	8,8,11,1,4,16,1,1,3,0,6,4,8	VACC2	Hospital E	Biofilm	2014
A198	6	461	+	Gt10(93)	9,8,6,2,4,13,3,4,10,3,10,4,8	VACC11	Hospital F	Biofilm	2014

[1]Sg, Serogroup; Mab, monoclonal subtype. NA: Not available. AQU: Al-Quds University

[3] MLVA-8(12) profile allele order: *Lpms1, Lpms3. Lpms13, Lpms17, Lpms19, Lpms31, Lpms33, Lpms34, Lpms35, Lpms38, Lpms39, Lpms40, Lpms44*

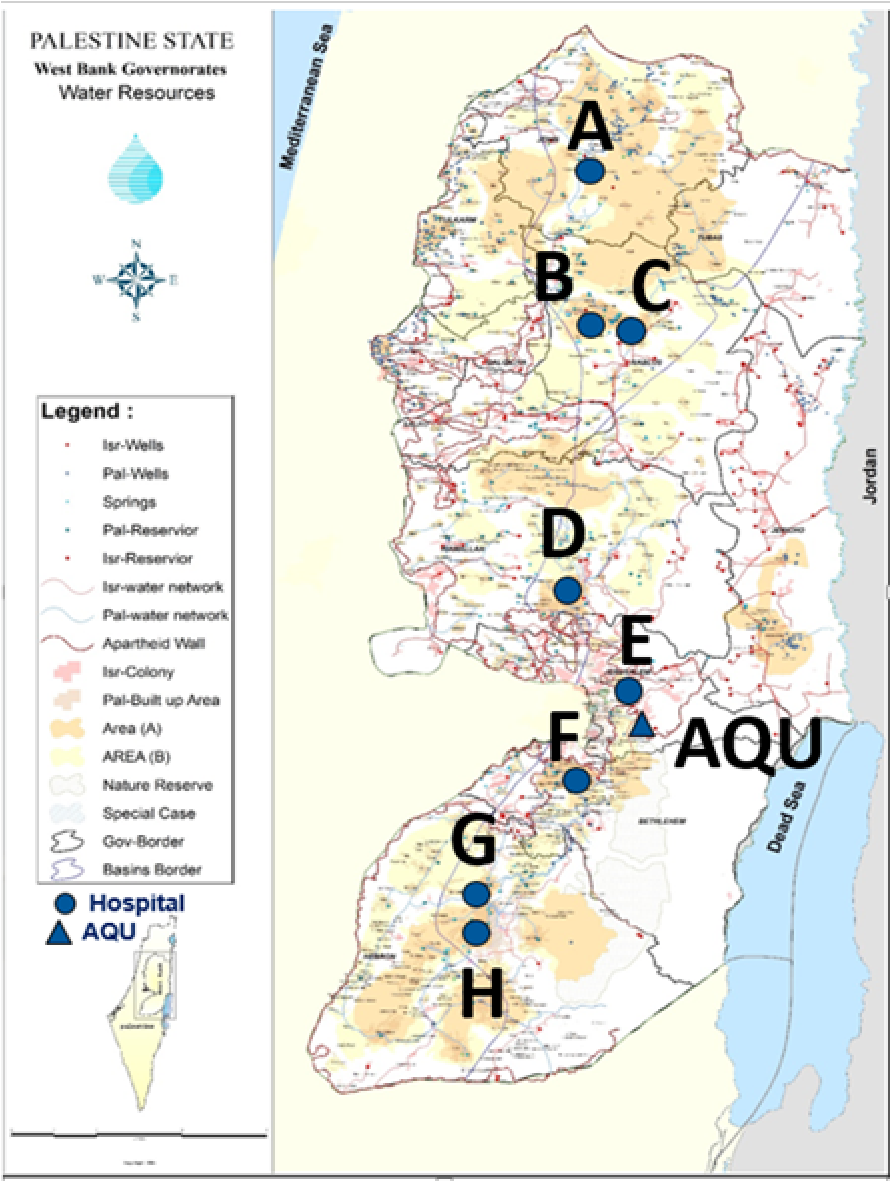

Figure S1: Sampling map of the eight hospitals and Al-Quds University in the West Bank

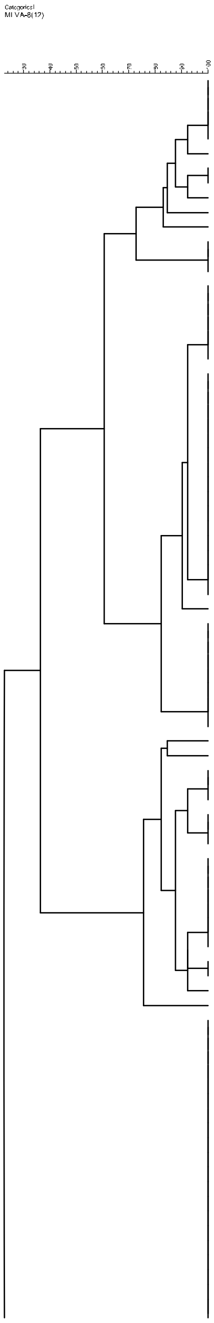

MLVA-8(12)

Lpms1	Lpms3	Lpms13	Lpms17	Lpms19	Lpms31	Lpms33	Lpms34	Lpms35	Lpms38	Lpms39	Lpms40	Lpms44	Key	Sg-Mab	ST (SBT)	MLVA-8 (12)	MLVA_CC	Sample type
9	8	8	2	4	13	2	2	18	3	10	4	8	A23	(2-14)	1438	Gt 16 (1)	VACC5	Biofilm
9	8	8	2	4	13	2	2	18	3	10	4	8	A34	(2-14)		Gt 16 (1)	VACC5	Biofilm
9	8	8	2	4	13	2	2	18	3	10	4	8	A38	(2-14)		Gt 16 (1)	VACC5	Biofilm
9	8	8	2	4	13	2	2	18	3	10	4	8	A46	6 Dresden		Gt 16 (1)	VACC5	Biofilm
9	8	8	2	4	13	2	2	18	3	10	4	8	A50	(2-14)		Gt 16 (1)	VACC5	Water
9	8	8	2	4	13	2	2	18	2	10	4	8	A36	(2-14)		Gt 16 (6)	VACC5	Biofilm
10	8	8	2	4	13	2	2	18	2	10	4	8	A91	(2-14)	1482	Gt 8 (7)	VACC5	Biofilm
10	8	8	2	4	13	2	2	18	2	10	4	8	A92	(2-14)	1482	Gt 8 (7)	VACC5	Biofilm
10	8	8	2	4	13	2	2	18	3	10	4	8	A166	(2-14)		Gt 8 (142)	VACC5	Biofilm
10	8	8	2	4	13	2	2	18	3	14	4	8	A24	1		Gt 8 (23)	VACC5	Biofilm
9	8	8	2	4	13	2	2	18	2	10	5	8	A94	(2-14)		Gt 16 (3)	VACC5	Biofilm
8	8	9	2	4	13	2	2	21	3	10	4	8	A193	6 Dresden		Gt 40 (47)	VACC5	Biofilm
8	8	9	2	4	13	2	2	21	3	10	4	8	A194	6 Dresden		Gt 40 (47)	VACC5	Biofilm
8	8	9	2	4	13	2	2	21	3	10	4	8	A195	6 Dresden		Gt 40 (47)	VACC5	Biofilm
9	8	6	2	4	13	3	4	10	3	10	0	8	A110	6 Dresden		Gt 10 (141)	VACC11	Biofilm
9	8	6	2	4	13	3	4	10	3	10	0	8	A116	6 Dresden		Gt 10 (141)	VACC11	Biofilm
9	8	6	2	4	13	3	4	10	3	10	0	8	A148	6 Dresden		Gt 10 (141)	VACC11	Water
9	8	6	2	4	13	3	4	10	3	10	0	8	A149	6 Dresden		Gt 10 (141)	VACC11	Biofilm
9	8	6	2	4	13	3	4	10	3	10	0	8	A157	6 Dresden		Gt 10 (141)	VACC11	Biofilm
9	8	6	2	4	13	3	4	10	3	10	0	8	A159	6 Dresden		Gt 10 (141)	VACC11	Biofilm
9	8	6	2	4	13	3	4	10	3	10	4	8	A108	6 Dresden		Gt 10 (93)	VACC11	Biofilm
9	8	6	2	4	13	3	4	10	3	10	4	8	A112	6 Dresden		Gt 10 (93)	VACC11	Biofilm
9	8	6	2	4	13	3	4	10	3	10	4	8	A114	6 Dresden		Gt 10 (93)	VACC11	Water
9	8	6	2	4	13	3	4	10	3	10	4	8	A115	6 Dresden		Gt 10 (93)	VACC11	Biofilm
9	8	6	2	4	13	3	4	10	3	10	4	8	A127	6 Dresden		Gt 10 (93)	VACC11	Biofilm
9	8	6	2	4	13	3	4	10	3	10	4	8	A128	6 Dresden		Gt 10 (93)	VACC11	Biofilm
9	8	6	2	4	13	3	4	10	3	10	4	8	A152	6 Dresden		Gt 10 (93)	VACC11	Biofilm
9	8	6	2	4	13	3	4	10	3	10	4	8	A153	6 Dresden		Gt 10 (93)	VACC11	Biofilm
9	8	6	2	4	13	3	4	10	3	10	4	8	A182	6 Dresden		Gt 10 (93)	VACC11	Biofilm
9	8	6	2	4	13	3	4	10	3	10	4	8	A183	6 Dresden		Gt 10 (93)	VACC11	Biofilm
9	8	6	2	4	13	3	4	10	3	10	4	8	A19	(2-14)	461	Gt 10 (93)	VACC11	Biofilm
9	8	6	2	4	13	3	4	10	3	10	4	8	A198	(2-14)		Gt 10 (93)	VACC11	Biofilm
9	8	6	2	4	13	3	4	10	3	10	4	8	A21	(2-14)	461	Gt 10 (93)	VACC11	Water
9	8	6	2	4	13	3	4	10	3	10	4	8	A71	(2-14)	461	Gt 10 (93)	VACC11	Biofilm
9	8	6	2	4	13	3	4	10	3	10	4	8	A97	(2-14)		Gt 10 (93)	VACC11	Biofilm
9	8	6	2	4	13	3	4	10	3	10	4	8	A99	6 Dresden		Gt 10 (93)	VACC11	Biofilm
9	8	6	2	4	13	3	0	10	3	10	4	8	A121	6 Dresden		Gt 55 (94)	VACC11	Biofilm
9	8	11	2	4	13	3	4	17	3	10	4	8	A107	(2-14)		Gt 9 (92)	VACC11	Biofilm
9	8	11	2	4	13	3	4	17	3	10	4	8	A138	6 Dresden		Gt 9 (92)	VACC11	Biofilm
9	8	11	2	4	13	3	4	17	3	10	4	8	A177	(2-14)		Gt 9 (92)	VACC11	Biofilm
9	8	11	2	4	13	3	4	17	3	10	4	8	A178	6 Dresden		Gt 9 (92)	VACC11	Biofilm
9	8	11	2	4	13	3	4	17	3	10	4	8	A22	(2-14)	461	Gt 9 (92)	VACC11	Biofilm
9	8	11	2	4	13	3	4	17	3	10	4	8	A82	(2-14)	461	Gt 9 (92)	VACC11	Biofilm
9	8	11	2	4	13	3	4	17	3	10	4	8	A84	(2-14)		Gt 9 (92)	VACC11	Biofilm
9	8	11	2	4	13	3	4	17	3	10	4	8	A86	(2-14)		Gt 9 (92)	VACC11	Biofilm
8	8	11	1	0	16	1	1	3	3	6	4	8	A8	6	187	Gt 84 (106)	VACC2	Biofilm
8	8	11	2	0	16	1	1	3	0	6	4	8	A93	(2-14)		Gt 24 (68)	VACC2	Biofilm
8	8	11	2	4	16	1	1	3	2	6	4	8	A144	1		Gt 64 (72)	VACC2	Biofilm
8	8	11	2	4	16	1	1	3	2	6	4	8	A95	6 Dresden		Gt 64 (72)	VACC2	Biofilm
8	8	11	2	4	16	1	1	3	2	6	4	8	A98	6 Dresden		Gt 64 (72)	VACC2	Biofilm
8	8	11	2	4	16	1	1	3	3	6	4	8	A119	6 Dresden		Gt 64 (74)	VACC2	Biofilm
8	8	11	2	4	16	1	1	3	3	6	4	8	A129	6 Dresden		Gt 64 (74)	VACC2	Biofilm
8	8	11	2	4	16	1	1	3	3	6	4	8	A156	6 Dresden		Gt 64 (74)	VACC2	Biofilm
8	8	11	1	4	16	1	1	3	2	6	4	8	A37	(2-14)	1326	Gt 13 (72)	VACC2	Biofilm
8	8	11	1	4	16	1	1	3	2	6	4	8	A41	(2-14)		Gt 13 (72)	VACC2	Biofilm
8	8	11	1	4	16	1	1	3	2	6	4	8	A44	(2-14)		Gt 13 (72)	VACC2	Biofilm
8	8	11	1	4	16	1	1	3	2	6	4	8	A68	(2-14)		Gt 13 (72)	VACC2	Biofilm
8	8	11	1	4	16	1	1	3	2	6	4	8	A69	(2-14)		Gt 13 (72)	VACC2	Biofilm
8	8	11	1	4	16	1	1	3	2	6	4	8	A70	(2-14)		Gt 13 (72)	VACC2	Biofilm
8	8	11	1	4	16	1	1	3	2	6	4	8	A79	(2-14)	1326	Gt 13 (72)	VACC2	Biofilm
8	8	11	1	4	16	1	1	3	0	6	4	8	A196	(2-14)		Gt 13 (143)	VACC2	Biofilm
8	8	11	1	4	16	1	1	3	0	6	4	8	A197	10		Gt 13 (143)	VACC2	Biofilm
8	8	11	1	4	16	1	1	3	3	6	4	8	A10	6		Gt 13 (106)	VACC2	Biofilm
8	8	6	2	4	8	1	1	3	2	6	4	8	A100	1		Gt 38 (109)	VACC2	Biofilm
7	7	10	2	4	9	4	2	18	3	14	5	5	A130	1		Gt 6 (18)	VACC1	Biofilm
7	7	10	2	4	9	4	2	18	3	14	5	5	A131	1		Gt 6 (18)	VACC1	Biofilm
7	7	10	2	4	9	4	2	18	3	14	5	5	A132	1		Gt 6 (18)	VACC1	Biofilm
7	7	10	2	4	9	4	2	18	3	14	5	5	A133	1		Gt 6 (18)	VACC1	Biofilm
7	7	10	2	4	9	4	2	18	3	14	5	5	A134	1		Gt 6 (18)	VACC1	Biofilm
7	7	10	2	4	9	4	2	18	3	14	5	5	A135	1		Gt 6 (18)	VACC1	Biofilm
7	7	10	2	4	9	4	2	18	3	14	5	5	A162	1		Gt 6 (18)	VACC1	Biofilm
7	7	10	2	4	9	4	2	18	3	14	5	5	A163	1		Gt 6 (18)	VACC1	Biofilm
7	7	10	2	4	9	4	2	18	3	14	5	5	A164	1		Gt 6 (18)	VACC1	Biofilm
7	7	10	2	4	9	4	2	18	3	14	5	5	A165	1		Gt 6 (18)	VACC1	Biofilm
7	7	10	2	4	9	4	2	18	3	14	5	5	A167	1		Gt 6 (18)	VACC1	Biofilm
7	7	10	2	4	9	4	2	18	3	14	5	5	A168	1		Gt 6 (18)	VACC1	Biofilm
7	7	10	2	4	9	4	2	18	3	14	5	5	A180	1		Gt 6 (18)	VACC1	Biofilm
7	7	10	2	4	9	4	2	18	3	14	5	5	A184	1		Gt 6 (18)	VACC1	Biofilm
7	7	10	2	4	9	4	2	18	3	14	5	5	A186	1		Gt 6 (18)	VACC1	Biofilm
7	7	10	2	4	9	4	2	18	3	14	5	5	A187	1		Gt 6 (18)	VACC1	Biofilm
7	7	10	2	4	9	4	2	18	3	14	5	5	A188	1		Gt 6 (18)	VACC1	Biofilm
7	7	10	2	4	9	4	2	18	3	14	5	5	A189	1		Gt 6 (18)	VACC1	Biofilm
7	7	10	2	4	9	4	2	18	3	14	5	5	A190	1		Gt 6 (18)	VACC1	Biofilm
7	7	10	2	4	9	4	2	18	3	14	5	5	A191	1		Gt 6 (18)	VACC1	Biofilm

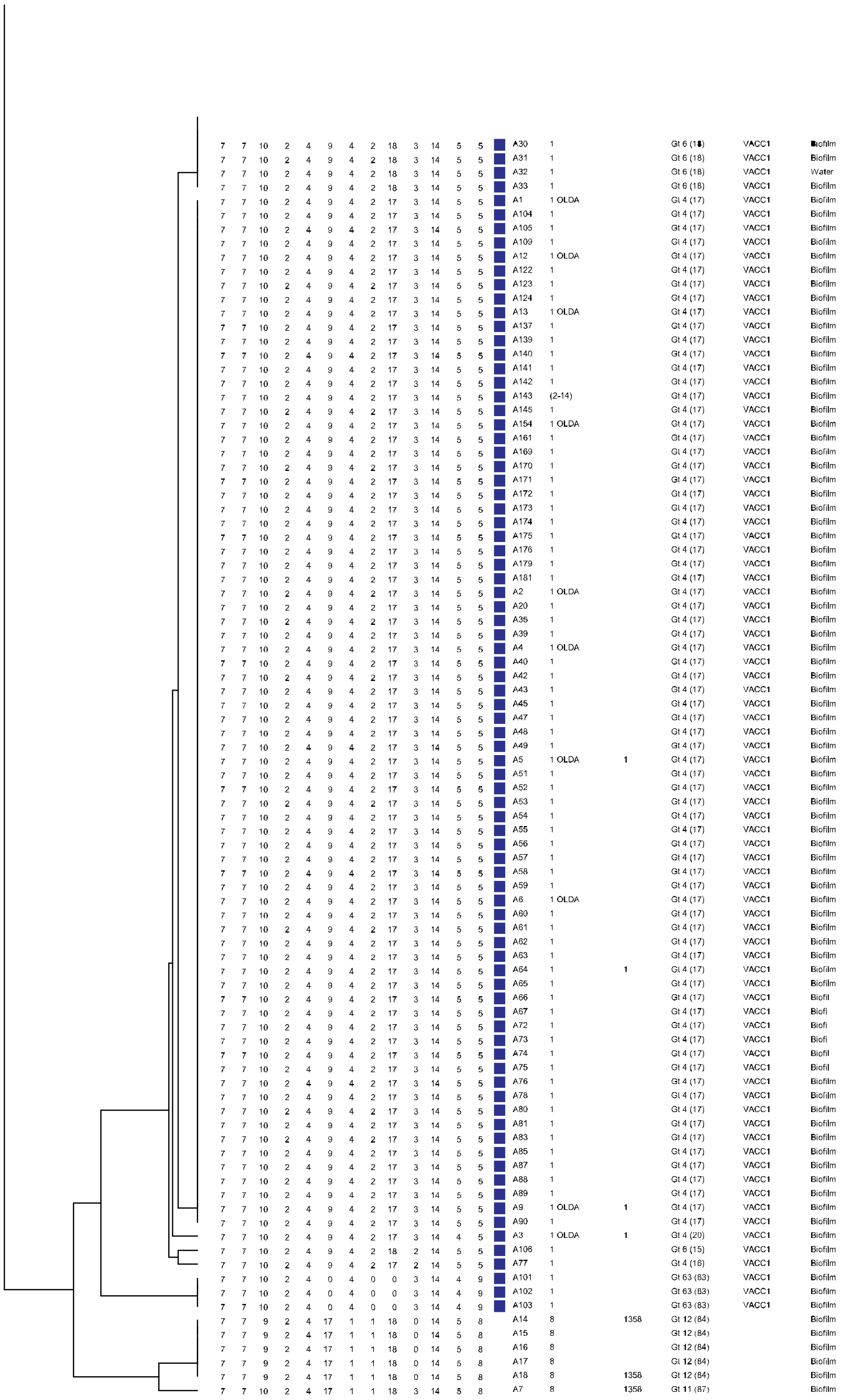

Figure S2: UPGMA based clustering analysis of the MLVA-8(12) profiles of 180 *L. pneumophila* strains isolated from water and biofilm samples of the Al-Quds University campus and eight hospitals of the West Bank. MLVA clusters (VACC) of three or more genotypes were defined using a cutoff of 60% of similarity and are shown in colors: VACC1, blue; VACC2, yellow; VACC5, green; VACC11, purple. (for details see Pecellín 2016 [37]).
